# Engineering a juxtamembrane-targeting CAR T-cell against mesothelin: a novel binder resilient to shed antigen for enhanced efficacy against ovarian and pancreatic cancer

**DOI:** 10.3389/fimmu.2026.1805950

**Published:** 2026-07-01

**Authors:** John Scholler, Ai Song, Mansi Deshmukh, Decheng Song, Khatuna Gabunia, Mei Ji, Shimin Liu, Ting-Jia Fan, Gayathri Gulendran, Carl H. June, Don L. Siegel

**Affiliations:** 1 Center for Cellular Immunotherapies, Perelman School of Medicine, University of Pennsylvania, Philadelphia, PA, United States; 2 Department of Pathology and Laboratory Medicine, Perelman School of Medicine University of Pennsylvania, Philadelphia, PA, United States; 3 Parker Institute for Cancer Immunotherapy at the University of Pennsylvania, University of Pennsylvania, Philadelphia, PA, United States

**Keywords:** adoptive cell therapy, chimeric antigen receptor, efficacy improvement, mesothelin, ovarian and pancreatic cancer, safety enhancement, solid tumors, therapeutic development

## Abstract

Mesothelin is an attractive target for CAR T therapy on a number of cancer types; however, the efficacy of this therapy is diminished because the bulk of the cell surface-expressed mesothelin is shed through naturally occurring proteolysis leaving behind a short juxtamembrane peptide “stump”. The two problems this creates are one, the bulk of the target protein is no longer on the tumor cell and two, soluble, shed mesothelin persists in the tumor microenvironment and circulates in blood and other body fluids, where it can bind mesothelin-targeted CAR T cells and act as a decoy that reduces engagement with tumor cell–surface mesothelin. These issues have contributed at least in part to the lack of desired efficacy in human clinical trials utilizing CAR T cells that target membrane distal regions of mesothelin (i.e., the shed domain) such as those utilizing the variable domains of anti-mesothelin monoclonal antibodies SS1 and M5. In addition, there have been safety concerns regarding the targeting of mesothelin on normal tissues. Here we describe CAR T cells that utilize novel phage display-derived antibodies specific for the mesothelin stump domain, thus being unaffected by the natural process of mesothelin shedding. Mesothelin “stump-specific” CAR T cells (CAR 422) had cytotoxicity and *in vivo* activity that were comparable to previously studied anti-mesothelin CAR T cells. Importantly, CAR 422 T cells were effective against tumor cells that were resistant to conventional anti-mesothelin CAR T cells and showed reduced on-target/off tumor toxicity in a human mesothelin knock-in mouse model. Thus, CAR 422 holds potential as a next-generation therapy for challenging solid tumors.

## 1 Introduction

Mesothelin (MSLN) is a glycosylphosphatidylinositol (GPI)-anchored cell surface protein usually restricted to mesothelial cells but is overexpressed in multiple solid tumors, including ovarian and pancreatic cancers (1–3). Its tumor-specific expression and limited presence in normal tissues make it an attractive target for antibody and chimeric antigen receptor (CAR) T-cell therapy (4–16).

However, development of effective anti-mesothelin CAR T cells has been challenged by dense stroma (15, 17), the targeting of membrane distal epitopes whose sites compete with endogenous proteins like MUC16 (18), and the presence of shed mesothelin in the tumor microenvironment (19). Soluble mesothelin can act as a decoy, binding distal targeting CARs such as those expressing the SS1 and M5 scFvs and hindering T-cell engagement with tumor cells (19–21). To overcome this, Liu et al. engineered CAR T cells targeting a juxtamembrane epitope near the membrane protease cleavage site of mesothelin using the mouse-derived 15B6 antibody (22). These CAR T cells retained activity in the presence of shed antigen and exhibited potent antitumor efficacy in preclinical models. In a subsequent study, a humanized version (h15B6) demonstrated marked activity against pancreatic cancer xenografts, overcoming resistance observed with shed-binding CARs (23). Concurrently with the initiation of a clinical trial evaluating h15B6 CAR T cells in patients with mesothelin-expressing solid tumors (www.clinicaltrials.gov, NCT06885697), these investigators sought alternative MSLN juxtamembrane-targeting domain antibodies through the immunization of rabbits (24). Given the potential utility of CAR T cells targeting non-shed juxtamembrane epitopes of MSLN, we describe an alternative approach that provided a large array of “stump-binding” scFvs by using a 40-billion-member combination naïve/non-immune canine antibody phage display library. Such an approach obviated the need for animal immunization with human MSLN and was therefore unaffected by natural self-tolerance mechanisms that would have occurred in previous studies (22, 24) given the high degree of homology between the juxtamembrane domain of human MSLN and those of mouse and rabbit. Utilizing this approach, several dozen MSLN stump binding scFvs were isolated. We detail the design, functional characterization, antitumor efficacy, and attenuated on-target/off-tumor activity of a lead candidate designated, CAR 422, and highlight its potential to overcome therapeutic limitations of MSLN-directed CAR T cells previously evaluated in human clinical trials.

## 2 Results

### 2.1 Isolation and characterization of scFvs targeting membrane proximal epitopes of mesothelin

**Figure 1:**
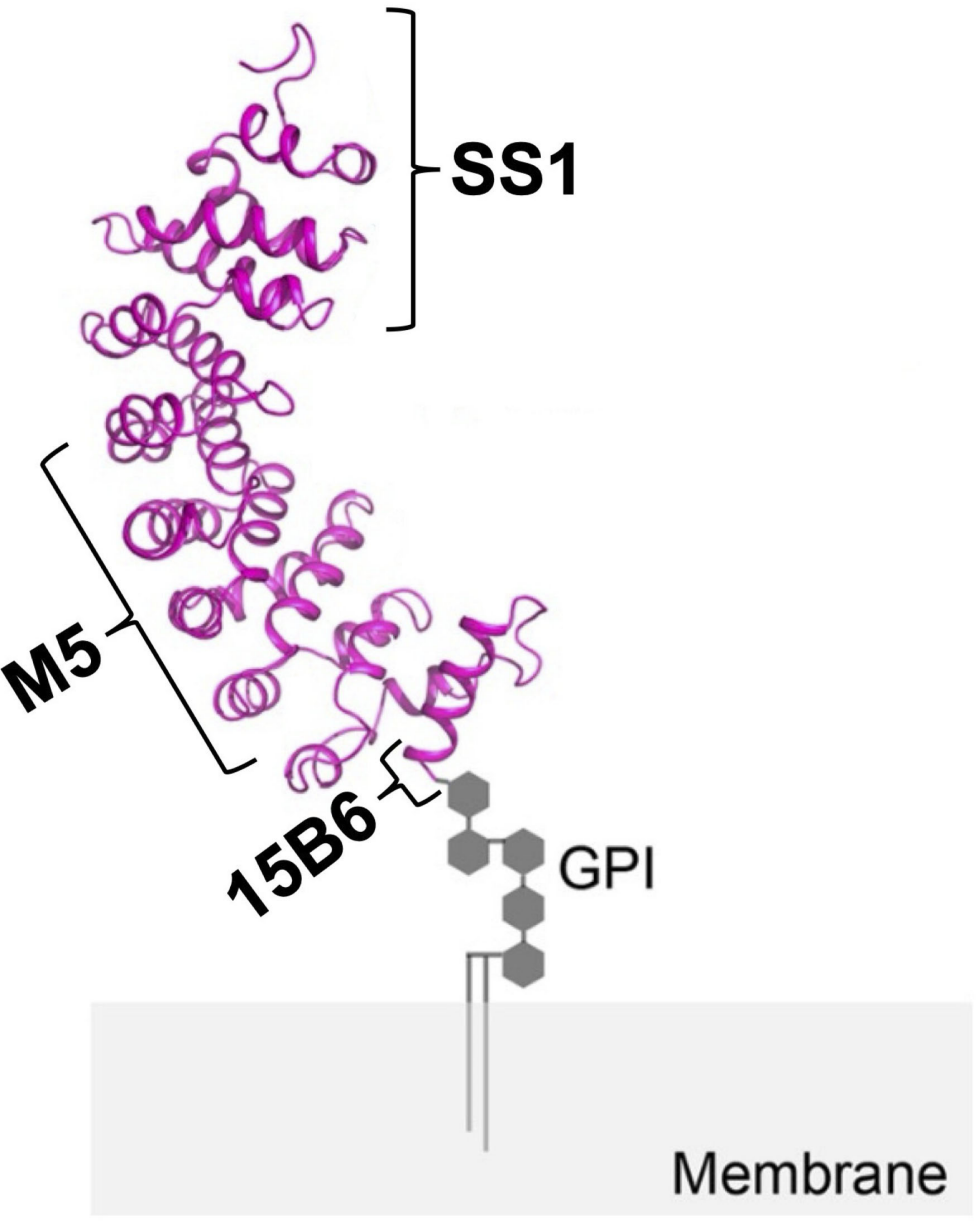
Schematic of mesothelin. Cartoon showing mesothelin and its GPI linkage, and binding sites for SS1, M5, and 15B6 antibodies. SS1 and M5 bind at sites distant from the MSLN proteolytic cleavage sites and can also bind to shed MSLN. The 15B6 scFv binds to cell-associated MSLN stump near the plasma membrane which remains after MSLN cleavage. Modified from Ma et al. (25).

**Figure 2:**
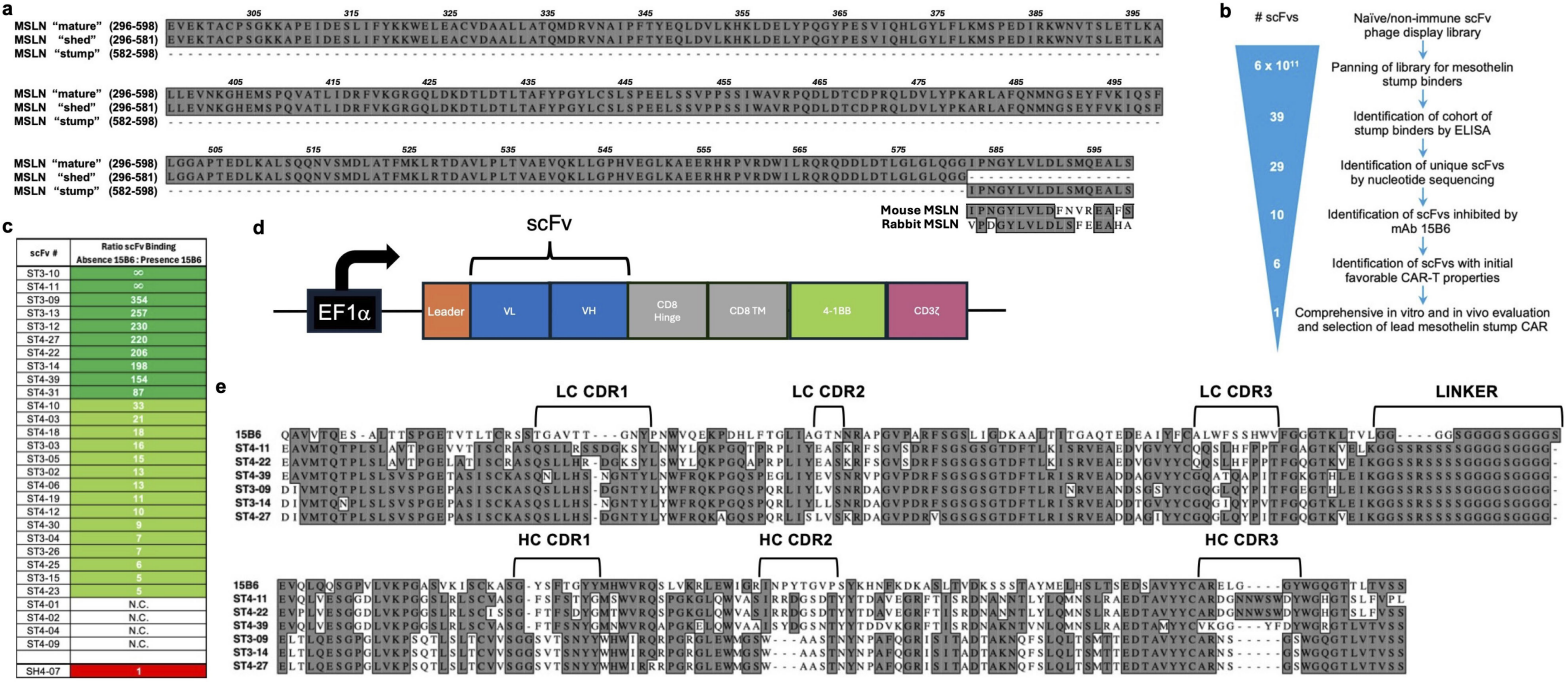
Isolation and characterization of scFvs targeting membrane proximal epitopes of mesothelin. **(a)** Amino acid alignment of mature, shed, and stump regions of human MSLN proteins. Homology of murine (Genbank AAH23753.1) and rabbit (NCBI Reference Seq XM_070064180.1) MSLN proteins to the human stump region is illustrated. **(b)** Canine B cell-derived scFv naïve/non-immune phage display library screening strategy. Positive/negative panning on full-length MSLN and shed-length MSLN proteins, respectively, enriched for scFvs targeting stump region of MSLN. Twenty-nine unique stump binders identified by nucleic acid sequencing from an initial cohort of 39 were further characterized as scFvs and a subset as T cell chimeric antigen receptors. **(c)** Competition ELISA assay used to rank ability of mAb15B6 reference MSLN stump-directed scFv to block binding of each of 29 stump-directed canine scFvs. Ratios of canine scFv binding to MSLN in the absence and presence of 15B6 are tabulated. The top 10 blocked scFvs (dark green shading) were chosen to be further screened as chimeric antigen receptors in order to narrow down the number of candidates for comprehensive evaluation to a total of 6. For 4 of the 29 scFvs, ELISA signals were too low to confidently determine binding ratios (“N.C.”). ScFv SH4-07, a scFv from library panning determined to be specific for the shed region of MSLN, served as a negative control for assay displaying equal binding to full-length MSLN whether 15B6 was present or not (ratio = 1). **(d)** Schematic of CAR construct inserted downstream of an EF1α-derived promoter within a 3rd-generation lentiviral vector plasmid comprising a CD28 transmembrane domain and 4-1BB and CD3ζ signaling domains. **(e)** Amino acid alignment of 15B6 reference MSLN stump-directed scFv with 6 canine stump-directed scFvs evaluated CARs in this study. All scFvs were used in the variable light chain/variable heavy chain (VL-VH) orientation. The 15B6 scFv sequence and linker were used as described by Liu et al. (22). The linker for canine scFvs was based on the pComb3X phage display vector system (30). Light chain and heavy chain CDRs were assigned according to IMGT nomenclature and identified using the Domain-GapAlign tool (55).

Membrane-bound mature mesothelin (MSLN) protein expresses binding sites on its membrane distal and central domains for antibodies SS1 (18, 25) and M5 (26), respectively, in addition to a juxtamembrane binding site for the 15B6 antibody (22) (**Figure 1**). MSLN is initially derived from a 622-amino acid precursor protein which subsequently undergoes furin cleavage releasing the soluble N-terminal 295-amino acid megakaryocyte potentiating factor resulting in a 303-amino acid mature form of the protein (amino acids 296-598) attached to the cell surface through a GPI linkage (22, 27, 28). Mesothelin can be shed from the cell membrane through the action of proteolytic enzymes with major cleavage sites within a carboxy terminal peptide comprising amino acids 582 to 598 (22, 23, 29). This results in the release of the bulk of mesothelin beginning at residue 296 and extending to approximately residue 581, and retention of a membrane-bound mesothelin “stump domain” beginning around residue 582 and extending to residue 598 (**Figure 2a**).

Antibody binding fragments specific to the stump region of MSLN were isolated from a canine scFv naïve/non-immune IgM/IgG/λ/κ phage display library containing an estimated 40 billion individual randomly recombined light and heavy chain variable domains constructed from canine B cell mRNA and the pComb3X phagemid vector as described (30–32) using oligonucleotide primers based on published canine immunoglobulin heavy and light chain germline genes (33). The use of such an antibody phage display library provides an enhanced repertoire of scFvs unbiased by natural self-tolerance mechanisms and immune selection when compared with a more limited diversity of antibodies that would be expected from immunized mice (22) or rabbits (24) where homology to endogenous MSLN stump domain is high (71% and 65% respectively) (**Figure 2a**). In our experience, because canine immunoglobulin genes show relatively high homology to human counterparts, scFvs isolated from this library have been useful in dozens of antibody selection campaigns not only for veterinary clinical trials but for human application including use of a canine phage library-derived scFv in CAR T cells being evaluated in a human clinical trial (www.clinicaltrials.gov, NCT06544265).

To isolate scFvs to the stump region of MSLN, rather than pan the scFv phage display library against the isolated 17-amino acid stump peptide such as that used for immunization of mice (22) or rabbits (24), the scFv phage display library was panned against immobilized full-length recombinant human MSLN protein after negative selection against a recombinant form of human MSLN representing shed MSLN. Selection of scFvs in this manner would seek to preserve the native structure of the stump portion of MSLN enabling the capture of scFvs directed to putative conformational epitopes that might not be preserved by an isolated 17-amino acid peptide. Negative selection to shed domains was achieved through two means: (1), pre-incubation of the phage library in streptavidin-coated microplate wells with immobilized biotinylated MSLN (296-580) with subsequent retrieval of non-absorbed phage particles and (2), addition of soluble non-biotinylated MSLN (296-580) to the non-absorbed phage particles to serve as a competitor during positive selection in wells coated with streptavidin and full-length (296-598) recombinant biotinylated MSLN.

Starting with ~600 billion scFv phage clones and after 4 rounds of negative/positive selection, ~400 million potential stump binders were isolated from panning round outputs. Sixty-six scFv phage clones were randomly selected from panning rounds 3 and 4 (clone nomenclature comprising “PX-Y” where “X” is panning round and Y is clone number), produced as monoclonal phage-displayed scFvs, and examined by phage ELISA for binding to the truncated and full-length forms of MSLN (**Supplementary Figure 1**).

Of the 66 randomly selected scFv phage clones, 39 bound to full-length MSLN but not truncated MSLN suggesting specificity for the stump domain which is present in the long form but not the short form (**Figure 2b**). Of these 39 presumed “stump-binders”, nucleotide sequencing of the scFv heavy and light chain variable regions revealed 29 unique scFvs. Of the remaining 27 (of 66) randomly selected scFv phage clones, 26 bound to both long and truncated MSLN suggesting specificity to the shed region of MSLN, and 1 clone bound to neither. Of these 26, 7 of them were unique scFvs based on nucleotide sequencing of heavy and light chains. Having binned scFvs to MSLN stump binders vs. shed MSLN binders, scFv clone names were modified by removing “P” and replacing with “ST” or “SH”, respectively, to represent “STump” or “SHed”.

To reduce the number of unique stump binding scFvs to be evaluated *in vitro* and *in vivo* as T cell chimeric antigen receptors, two sequential screenings were performed. First, the extent to which reference murine mAb 15B6 could inhibit the binding of each of the 29 scFvs to full-length mesothelin was determined to assess stump epitope specificity. To develop this assay, a recombinant form of 15B6 was produced as a full-length murine IgG_1_ using the published amino acid sequences for the 15B6 heavy and light chains (22).

After verifying that recombinant 15B6 retained its binding to the mesothelin stump domain (**Supplementary Figure 2**), microplate wells coated with full-length MSLN were preincubated (or not) with an amount of 15B6 10-fold that which would be necessary to saturate the MSLN coating the wells. Phage displayed scFvs were then applied to MSLN-coated wells (in the presence or absence of 15B6) and after washing away unbound phage, bound phage were detected using an HRP-labeled anti-M13 phage secondary reagent (**Supplementary Figure 3**). In this experiment, the molar ratios of 15B6 to scFvs were ~300,000:1 (Materials and Methods 4.4) to ensure that an scFv with a potentially higher affinity than 15B6 would still be blocked by 15B6.

For each scFv, the ratio of scFv binding in the absence and presence of 15B6 was calculated (**Figure 2c**) and the 10 (of 29) scFvs most strongly blocked by 15B6 were selected for further study in a second screening assay. A useful internal control was scFv P4-07 [which was determined to bind to shed MSLN, **Supplementary Figure 1**, and renamed SH4-07, (**Figure 2c**)] which showed a 15B6 inhibition ratio of unity as would be expected from a MSLN shed-directed scFv.

For the second screening assay, the 10 scFvs selected from the 15B6 inhibition assay were cloned into our platform CAR construct (**Figure 2d**) and then packaged into lentiviral particles for transduction of healthy normal donor (ND) human T cells. An *in vitro* killing assay using the human CAR T cells targeting MSLN expressed on GFP-transduced human cell lines AsPC1 (pancreatic ductal adenocarcinoma) and OVCAR8 (ovarian adenocarcinoma) was performed that allowed us to reduce our initial 10 MSLN stump binders down to 6 (**Supplementary Figure 4a**). Stump CAR T cells ST3-09, ST3-14, ST4-11, ST4-22, ST4–27 and ST4–39 were selected as those which showed robust killing by reduction of GFP signal (lack of tumor cells) over a range of E:T ratios as compared to non-transduced cells (NTD) and tumor alone.

Alignment of the predicted amino acid sequences for this group of 6 scFvs along with 15B6 reference scFv (**Figure 2e**) show that all 6 scFvs are quite distinct from 15B6. All utilize kappa isotype light chains. ST4–11 and ST4–22 show identical CDR2 and CDR3 regions in both heavy and light chains but have marked differences in their CDR1 regions as well as framework regions 1 and 4. ST3-09, ST3-14, and ST4–27 have nearly identical heavy chains (except for a single amino acid difference in framework 2 of ST4-27), but they are paired with light chains that show significant degrees of diversity in all framework and CDR regions. ST4–39 is distinctly different in primary structure from the other 5 scFvs (**Supplementary Table 1**).

### 2.2 Screening top six stump CARs

**Figure 3:**
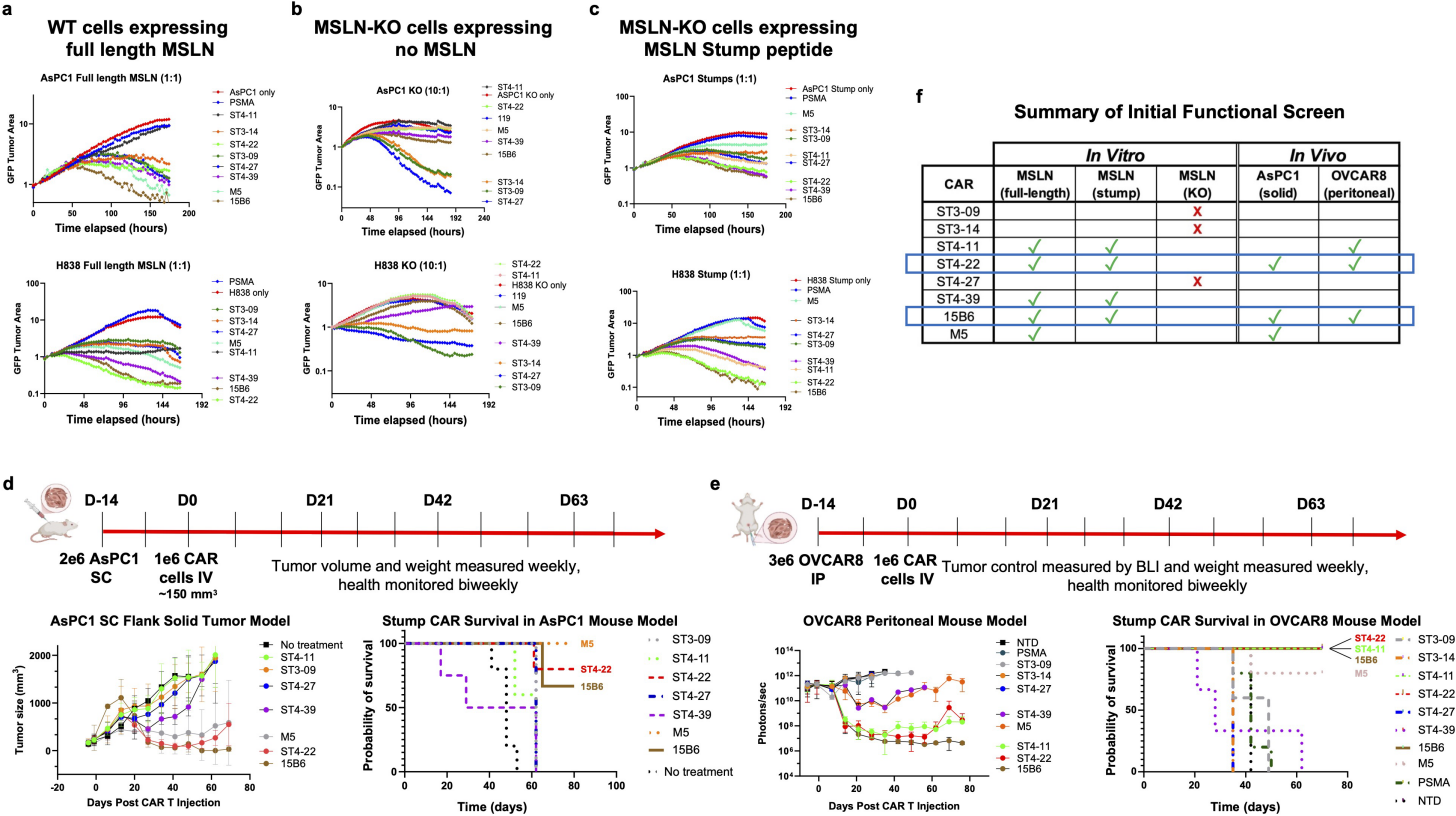
Stump CAR targeting and efficacy screen in healthy donor ND608 T cells. The cytotoxic *in vitro* activity of stump binding CAR T cells was assessed by titration at E:T ratios of 10:1, 3:1, 1:1, 0.3:1 and 0.1:1 as single values in 96-well plate co-cultures of tumor cell lines and evaluated by Incucyte^®^ live cell imaging. **(a)** The cytotoxic activity of each CAR T cell was titrated against AsPC1 and H838 wild-type cells. Only the 1:1 E:T ratio is shown to highlight differences. A qualitative evaluation was used to select best killing based on a diminished tumor area. Another donor showed similar results. **(b)** To eliminate stump binders with non-MSLN specific killing, CAR T cells were co-cultured with AsPC1 and H838 cell lines from which MSLN was knocked out (KO). Only the 10:1 ratio is shown where maximum off-target activity can be seen. For both cell lines, binders ST3-09, ST4–27 and ST3–14 revealed non-MSLN specific killing and were eliminated from further evaluation. **(c)** To assess stump specificity, CAR T cells were co-cultured with AsPC1 and H838 MSLN-KO cells expressing lentivirus-transduced GPI-linked stump peptide. To highlight differences, only 1:1 E:T ratio results are shown and reveal ST4-11, ST4-22, ST4-39, and 15B6 show specificity for MSLN stump peptide. The non-MSLN specific binders have similar activity at 1:1 as they did with wild type cells. Another T cell donor gave similar results. **(d)** Stump binders were assessed *in vivo* to determine rate of tumor clearance and the persistence of response in an AsPC1 flank solid tumor model in NSG mice with 5 mice per group. When 2e6 AsPC1 cells were injected subcutaneously into the right flank with Matrigel and reached ~150mm^3^ in volume (~14 days after implantation), 1e6 CAR^+^ T cells were injected IV. Tumor volumes were plotted as mean with SEM and survival as Kaplan-Meier curve. **(e)** Stump binders were assessed *in vivo* to determine rate of tumor clearance and the persistence of response in an OVCAR8 peritoneal metastasis model in NSG mice with 5 mice per group. Fourteen days after intraperitoneal injection of 3e6 OVCAR8 tumor cells expressing click beetle red (CBR) luciferase for *in vivo* monitoring, 1e6 CAR^+^ T cells were injected IV. Weekly tumor BLI is plotted as mean with SEM and survival plotted as Kaplan-Meier curve. **(f)** Summary of initial *in vitro* and *in vivo* functional screens of MSLN stump-directed CAR T cells.

The top 6 stump CAR scFv candidates were engineered into healthy human T cells for *in vitro* and *in vivo* evaluations to assess their abilities to specifically target cells expressing full length or just the MSLN stump domain and to control tumor growth of cells. In addition to AsPC1 and OVCAR8 cells, a third cell line, H838 (non-small cell lung adenocarcinoma), was used in evaluations. The percent transduction of each stump CAR in ND608 donor T cells and their surface levels of expression (MFI) are shown in **Supplementary Figure 4b**. The performance of each stump CAR was compared relative to our conventional human MSLN CAR T comprising M5 scFv and the non-MSLN targeting human prostate-specific membrane antigen (PSMA) or human CD19 (CAR119) CARs (**Figure 3**). The M5, PSMA and CAR119 CARs have been previously described (26, 34, 35).

To determine relative efficacy and target specificity, each stump CAR was co-cultured for 7 days with GFP-expressing AsPC1 or H838 cell lines expressing either their normal full length MSLN (wild type, WT), no MSLN (MSLN-KO), or MSLN stump domain engineered into MSLN-KO cells (**Figures 3a–c**). Killing efficiency was measured by Incucyte^®^ live cell imaging every 3 h for changes in GFP intensity, which directly correlates to number of tumor cells surviving. All stump CAR T cells co-cultured with wild type AsPC1 cells impaired the tumor growth with only ST4–11 and the non-targeting PSMA CAR unable to show anti-tumor efficacy. Stump CAR T cells co-cultured with the wild type H838 cells which grow faster and are more sensitive to CAR T cell killing, were able to control tumor with a wider range of effectiveness. ST4-22, 15B6, ST4–39 and M5 had the ability to reduce the tumor numbers, while ST3-09, ST3-14, ST4-11, ST4–27 were only able to maintain steady tumor cell numbers (**Figure 3a**). These results indicated that the stump CARs could control full length MSLN comparably to our standard CAR M5 that binds to an epitope distal to the stump epitope (26).

To confirm the specificity of the stump CARs to the stump region of MSLN GPI-linked surface expressed protein, we first knocked out MSLN expression (MSLN-KO) in both the AsPC1 and H838 cell lines by CRISPR technology. These MSLN-KO cells were then used to identify CAR T cells that could kill tumor cells independent of stump targeting. In addition, to confirm that stump CAR T cell killing was directed specifically to the stump portion of MSLN, we transduced these MSLN-KO cell lines with a lentiviral construct encoding the 582–598 amino acid membrane proximal stump region which included a 3x FLAG tag on the amino terminal end for detection.

To identify non-MSLN stump CAR T cell activity, we co-cultured the anti-stump candidate CAR T cells with AsPC1 or H838 MSLN-KO cells at a high 10:1 E:T ratio to increase the ability to detect off-target killing. CAR T cells expressing the ST3-09, ST3–14 and ST4–27 scFv had non-specific killing activity against both cell lines, eliminating these as viable scFvs (**Figure 3b**). Stump CARs co-cultured with AsPC1 cells that expressed only the stump epitope showed that each of the stump CARs slowed tumor growth with ST4-22, ST4–39 and 15B6 showing the greatest reduction (**Figure 3c**, top). As expected, CAR M5 lost its ability to control tumor cells only expressing the stump epitope. Similarly, in H838 co-cultures, ST4–22 and 15B6 recognized the stump region the best, followed by ST4–11 and ST4-39 (**Figure 3c**, bottom).

In a separate experiment, we transduced our stump CARs and M5 into Jurkat NFAT-GFP reporter cell lines to evaluate their ligand dependent and independent signaling (**Supplementary Figure 4c**). The induction of NFAT signaling was evaluated on wild type or MSLN-KO H838 cells. Only stump ST4–22 and 15B6 CAR cells demonstrated ligand-dependent signaling, which was specific to MSLN-expressing cells, similar to our distal targeting M5 CAR cells. Interestingly, 15B6 and M5 but not ST4-22, showed a non-MSLN directed activation after 50 h. In addition, the activation decay of 15B6 and M5 appear to be slower than ST4–22 suggesting a more rapid rest down for ST4-22.

The stump CAR activities were next evaluated *in vivo* using the xeno-mouse models of the pancreatic AsPC1 flank solid tumor and the ovarian OVCAR8 peritoneal tumor. The tumor microenvironment (TME) of each of these models is quite different, as the subcutaneous flank model of AsPC1 will develop a relatively vascularized dense stroma with no ascites formation while the TME in the peritoneal metastatic model of OVCAR8 is a soft liquid-filled cavity with slow fluid exchange developing terminal ascites where shed MSLN can accumulate. We have evaluated the amount of shedding generated innately by both AsPC1 and OVCAR8 cell lines alone in culture, showing these two cell lines have the potential to produce similarly high levels of shed MSLN compared to other cell lines we tested (**Supplementary Figure 5**).

After 2 weeks of AsPC1 tumor engraftment to a defined volume (~150mm^3^), 1e6 stump CAR+ T cells were administered IV, and changes in tumor volume were measured weekly (**Figure 3d**). By 3 weeks, ST4-22, 15B6 and M5 CAR T cell-treated mice had potent antitumor effects, and most mice were able to eliminate visual detection of the tumor with long-term survival, with a few animals succumbing to xenogeneic graft-versus-host-disease. ST4–39 showed early antitumor effects but failed to have persistent control of tumor growth by 4 weeks. The tumor growth kinetics in individual mice treated with ST4-11, ST4-22, 15B6 and M5 CAR T cells are shown in **Supplementary Figure 6a**. Mice maintained their weight and remained in good health (**Supplementary Figure 6b**). All other stump CARs failed to show any signs of tumor reduction. Based on these results, ST4–22 was considered the leading candidate in the AsPC1 pancreatic tumor model.

The OVCAR8 tumor cell line is known to produce proteases such as ADAM10 and ADAM17, which cleave large amounts of cell surface MSLN (22, 29), producing a milieu of shed soluble antigen capable of blocking MSLN CAR T cells, reducing their ability to target surface antigen and kill tumor. Thus, the OVCAR8 peritoneal mouse model is suitable for evaluating the ability of the stump CAR T cells to target tumor cells caused by cleaved MSLN by engaging with cell surface-bound full length MSLN as well as the residual juxtamembrane MSLN stump remaining after proteolytic cleavage.

Two weeks after peritoneal implantation of OVCAR8 tumor cells expressing click beetle red (CBR) luciferase for *in vivo* monitoring, 1e6 stump CAR^+^ T cells were administered IV, and 2 weeks later only the stump CARs ST4-22, ST4–11 and 15B6 had potent antitumor efficacy, leading to 100% long-term survival (**Figure 3e**). The tumor growth kinetics in individual mice treated with ST4-11, ST4-22, 15B6 and M5 CAR T cells are shown in **Supplementary Figure 6c**. Mice maintained their weight and remained in good health (**Supplementary Figure 6d**). It is notable that CAR M5 was able to effectively control AsPC1 tumors in our previous experiments yet failed to control OVCAR8 tumors. **Figure 3f** summarizes the *in vitro* and *in vivo* screening of the top 6 stump CAR candidates. These results indicate ST4–22 as a top candidate having good efficacy in both tumor models, including the resistant OVCAR8 tumor, and with no background ligand-independent signaling activity and no non-specific killing of MSLN KO cells.

### 2.3 Validation of ST4–22 as lead anti-MSLN stump CAR scFv

#### 2.3.1 CAR T manufacturing and initial activation kinetics

Before further characterization of ST4–22 CAR T cells, we first minimized the potential for induction of anti-CAR T immune responses by eliminating internal ATG start sites from alternate reading frames encoded in the transgene that could produce translated peptides greater than 50 amino acids in length. By mutating ATG start sites while not changing the amino acid composition of the CAR, we generated a modified nucleotide sequence of ST4–22 which we now designate CAR 422 (**Supplementary Figure 7**; **Supplementary Table 2**).

**Figure 4:**
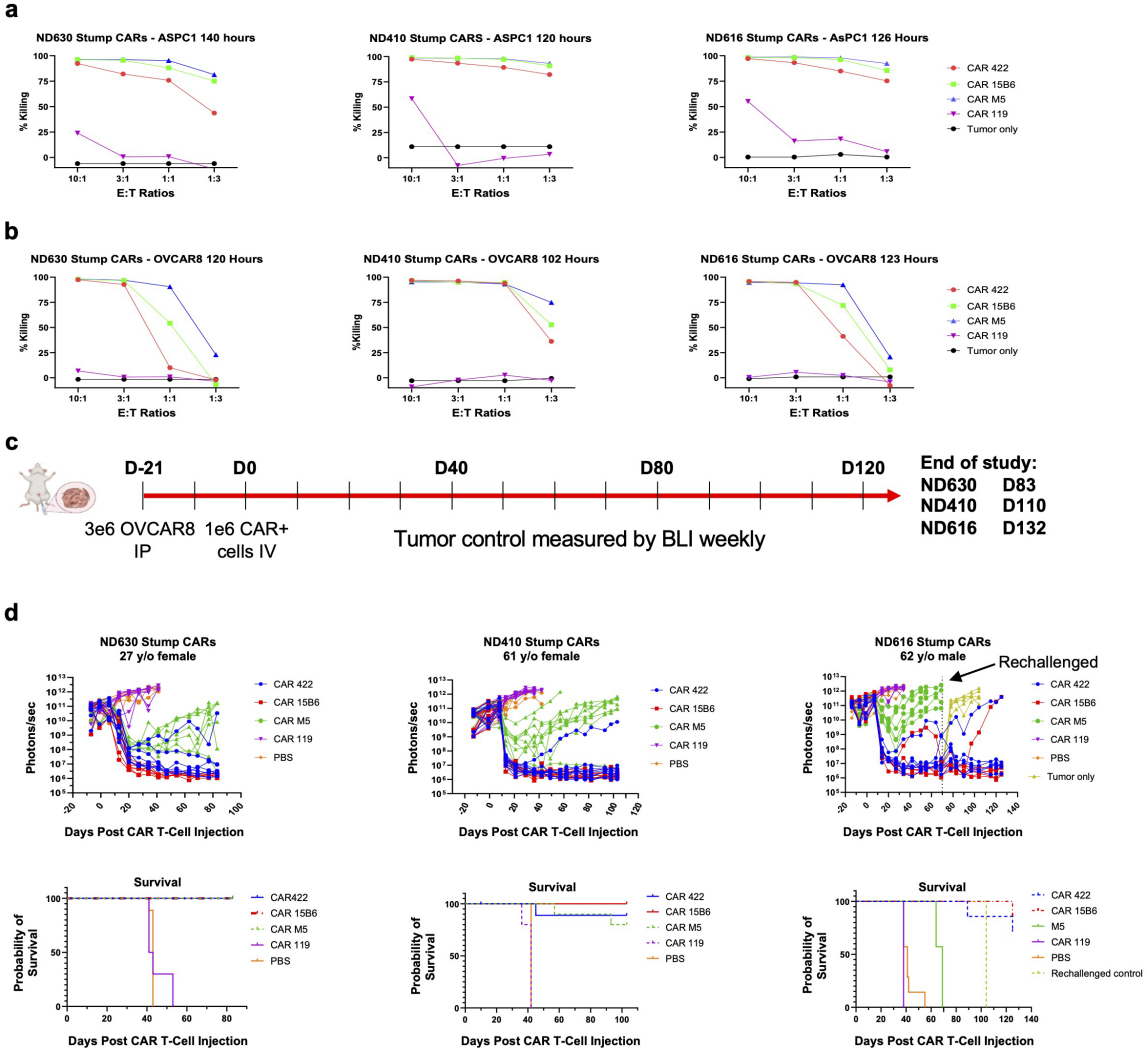
Killing efficacy of stump CARs *in vitro* and *in vitro* across multiple T cell donors. T cells from 3 healthy donors (ND630, ND410, ND616) varying in gender and age were transduced with MSLN stump-directed CAR 422 and CAR 15B6, along with distal MSLN targeting CAR M5 and CD19-targeting CAR 119 and compared for target cell killing *in vitro* and *in vivo*. CAR T cells were evaluated in Incucyte^®^ living imaging assays with co-cultures of **(a)** AsPC1 cells or **(b)** OVCAR8 cells at E:T ratios of 10:1, 3:1, 1:1 and 1:3 for the times indicated. Graphs were analyzed to show % killing. **(c)** Schema of CAR T cells evaluated *in vivo* in the OVCAR8 peritoneal mouse model. **(d)** CAR T cell tumor control for each of the three donors with associated mouse survival curves. ND630 CAR T cells were injected intravenously 14 days after the introduction of OVCAR8 tumor cells while ND410 and ND616 cells were injected 21 days afterwards. ND630 and ND616 CAR T groups had 7 mice/group while the ND410 groups had 10 mice/group. Treatment-naïve mice were rechallenged with tumor cells on Day 70 as control for tumor growth.

To evaluate MSLN stump-directed CAR 422 T cells under a number of *in vitro* and *in vivo* conditions, we engineered CAR 422 into 3 separate healthy T cell donors including both male and female and spanning 27 to 62 years of age. The manufacturing profiles of CAR 422 in the healthy donors ND630 (27 y/o female), ND616 (62 y/o male), and ND410 (61 y/o female) are similar in doubling rates and size compared to stump CAR 15B6 and control CARs M5 and 119 (**Supplementary Figure 8**). The killing ability of the CAR T cells from each of the 3 donors was first evaluated in *in vitro* co-cultures with either AsPC1 or OVCAR8 tumor cells at CAR T cell to tumor cell (E:T) ratios of 10:1, 3:1, 1:1 and 0.3:1. Targeting the AsPC1 cells, CAR 422 T cells had killing efficacy of greater than 75% except for the lowest E:T ratio of 1:3 in donor ND630. Overall, the efficacy of tumor control was slightly less than the stump CAR 15B6 and the distal targeting CAR M5 (**Figure 4a**). In the co-cultures with OVCAR8 tumor cells, CAR M5 maintained a stable tumor control down to a 1:1 ratio, while both stump CARs 422 and 15B6 lost tumor control at the 1:1 ratio for ND630 and ND616 and at the 1:3 ratio for ND410 (**Figure 4b**). In all co-cultures, CAR 422 showed a slightly lower efficacy profile compared to 15B6.

T cell cytokine production in co-cultures can indicate the unique signaling activity of a CAR T cell and also their relative cytotoxic strengths, especially the production of IFN-γ. For CAR T cells manufactured from ND410 and co-cultured with OVCAR8 tumor cells at 1:1 E:T ratio, stump CARs 422 and 15B6 produced similar levels of IFN-γ after 24 and 48hr while CAR M5 T cells had ~2 logs lower production of IFN-γ (~200,000 pg/mL vs. ~3,000 pg/mL) (**Supplementary Figure 9a**). Interestingly, TNF-α and IL-2 levels were comparable for M5, 422 and 15B6 CAR T cells. The IFN-γ levels correlate with OVCAR8 tumor control found *in vivo* indicating CAR M5 may be less activated in this model. Comparatively, co-culturing with the AsPC1 cells produced similar levels of IFN-γ, TNF-α, and IL-2 cells after 48hr with both the stump and non-stump anti-MSLN CARs (**Supplementary Figure 9b**). Together, IFN-γ production alone in a short-term assay may represent activation of CAR T cells and not a direct correlate of cell killing as that is driven mainly by a perforin/granzyme response.

The robustness of the CAR M5 in *in vitro* OVCAR8 co-cultures compared to the *in vivo* results shown in **Figure 3** probably reflects the time needed to build up the effective blocking concentrations of shed material in the tumor microenvironment. In the co-cultures, the shed material is likely just beginning to build up while in the OVCAR8 intraperitoneal mouse model, the shed material has already been accumulating for several weeks before CAR T cell treatment and may be more representative of a patient’s tumor microenvironment.

#### 2.3.2 Characterization of CAR 422 T cells in an OVCAR8 MSLN shedding mouse model

We next compared CAR422 T cells from these 3 normal T cell donors for *in vivo* efficacy with CARs 15B6 and M5 in our OVCAR8 tumor MSLN shedding mouse model (**Figure 4c**). As seen in our earlier stump CAR screening, CAR 422 effectively controls tumor to baseline levels by three weeks post stump CAR T cell administration (**Figure 4d**) while maintaining a healthy weight throughout the studies (**Supplementary Figure 10**). The lack of efficacy in distal targeting CAR M5 is also consistent with our earlier screening results. Stump CARs 422 and 15B6 T cells consistently have similar efficacy in tumor control and long-term survival.

We characterized the long-term robustness of CAR 422 tumor control by rechallenging the mice in the ND616 study by injecting an additional 3e6 OVCAR8 tumor cells IP on Day 70, 35 days after stable tumor control. Six out of the 7 CAR 422 mice and 7 out of the 7 CAR 15B6 mice after challenge with additional tumor maintained their tumor control to Day 104. One mouse in the 15B6 group showed signs of failure beginning at Day 97. To serve as a control for tumor growth in the absence of CAR treatment, 5 naïve mice were injected with OVCAR8 cells and showed rapid expansion in BLI to saturating levels in 10 days. This rechallenge experiment suggests immunosurveillance by stump-directed CAR T cells can provide rapid memory responses to effectively clear re-occurring tumor growth, indicating their potential as effective persistent therapeutics. To assess the level of soluble shed MSLN in the TME that may interfere with the activity of CAR T cells targeting membrane distal domains of MSLN, we measured soluble MSLN in the ascites fluid from 3 CAR119 and PBS control animals in the ND410 study that had developed ascites and were being euthanized 59 days post tumor implants. We also obtained 2 mice in the ND616 study that had developed ascites, one in the CAR119-treated control group and one in the CAR M5 group that needed to be euthanized 63 days post tumor implant. (**Supplementary Figure 11**). All sampled ascites fluids had soluble MSLN levels of greater than 10 ng/mL. The soluble MSLN levels were plotted against the individual mouse’s tumor BLI readings, which saturated out at 1e12 photons/sec. Interestingly, in the case of a CAR M5-treated mouse which showed slight tumor control, the levels were higher. As this is a single case, we cannot determine if the soluble MSLN level actually increased during antitumor response potentiated by the accumulation of dead tumor cell debris. In aggregate, we found levels of soluble MSLN in ascites fluid at 10 to 27 ng/mL that would be expected to inhibit the activity of CAR T cells targeting membrane distal domains of MSLN (22).

In a separate cohort of 3 mice per group, we evaluated the engraftment level of ND616 human T cells in the blood, spleen and peritoneal washes on Day 38 after intravenous injection, when tumors had been cleared and before the timing of the rechallenge. The ND616 CD45+T cells were not detected in the peripheral blood but only in the peritoneal cavity (**Supplementary Figure 12**). This indicates that the T cells effectively migrated into the peritoneum and remained around tumor cells in the highest numbers after intravenous injection. While the CAR 422 T cell group had similar numbers of CD45+ cells in ascites as CAR M5, the CAR M5 cells had lost their ability to control tumor, presumably due to cleavage of the targeted MSLN epitope and the presence of soluble shed MSLN. Interestingly, the spleens did not have residual T cells in all groups as assessed by flow cytometry and consistently had less splenomegaly in mice treated with CAR T cells targeting mesothelin (1.0 cm length) compared to the groups treated with non-targeting CAR 119 and the no treatment group (~1.6 cm length).

**Figure 5:**
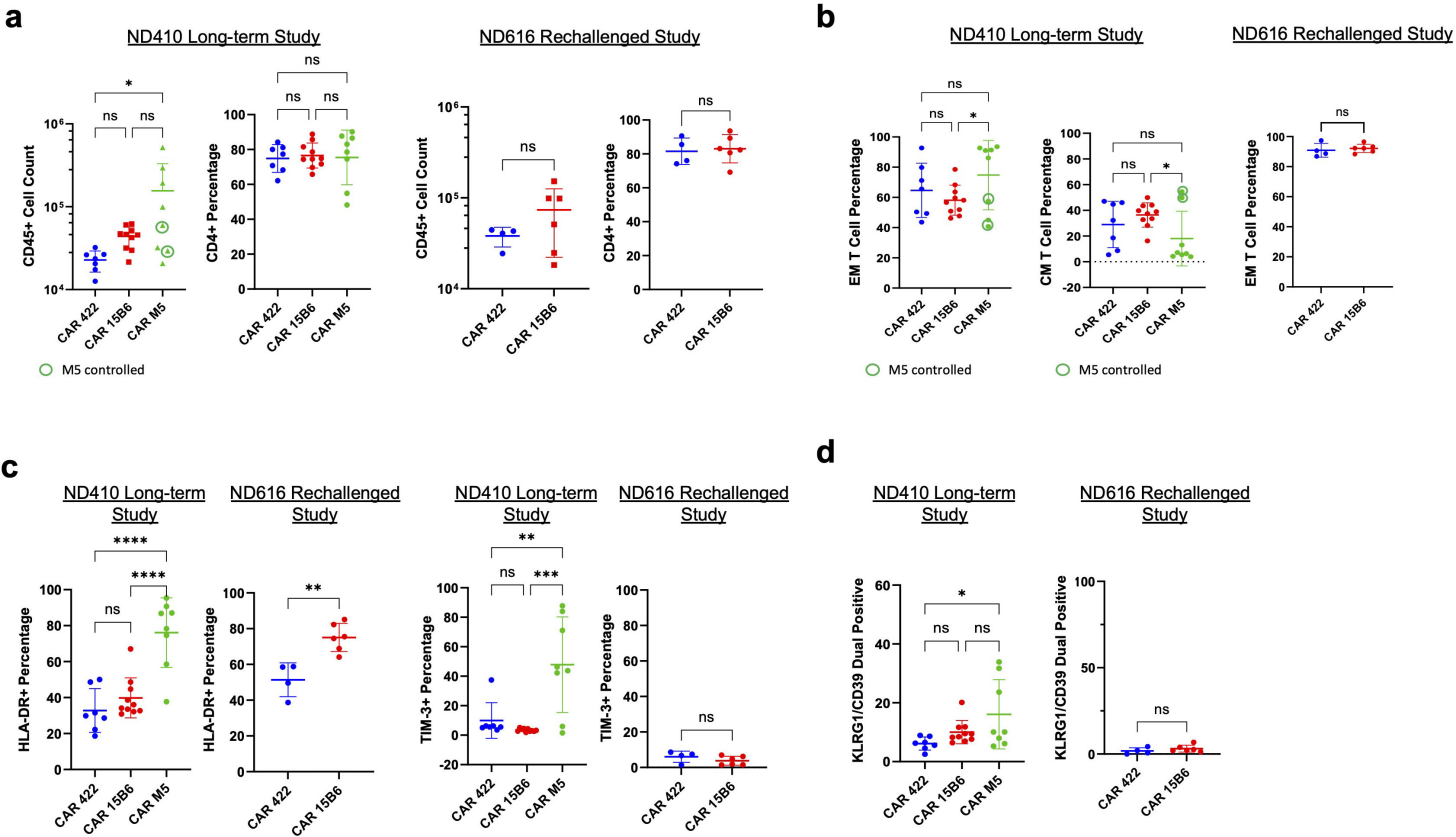
Phenotype and activation profile of peritoneal huCD45+ cells. **(a)** Total CD45+ cell count and %CD4+ T cells isolated from mouse peritoneum in ND410 long-term study and ND616 rechallenged study. Green circles indicate 2 mice from the CAR M5 group in which tumor was controlled in the long-term study. **(b)** CD62L x CD45RA memory phenotype of huCD45+ cells isolated in the long-term and rechallenged studies. Plotted are % effector memory (EM) cells (CD45RA- and CD62L-) and % central memory (CM) cells (CD45RA- and CD62L+). Green circles indicate 2 mice from the CAR M5 group in which tumor was controlled in the long-term study. **(c)** Activation status of peritoneal huCD45+ cells as defined by % HLA-DR positivity and %TIM-3 expression. **(d)** Exhaustion status of peritoneal huCD45+ cells as defined by KLRG1 and CD39 dual positivity. Statistical significance determined using Anova analysis for long-term evaluations and unpaired t test for rechallenge evaluations. *p<0.05, **p<0.003, ***p<0.0002, ****p<0.0001.

We then characterized the huCD45+ T cell levels isolated from the mouse ascites in the ND410 long-term study, 90 days after tumor control, and in the ND616 rechallenged study mice, 34 days after the rechallenge. In the ND410 long-term study, mice which controlled tumor with CARs 422, 15B6, and M5 had similar CD45+ cells levels while the levels of CD45+ cell numbers tended to be higher in the CAR M5 mice which could not control the tumor (**Figure 5a**). The CAR M5 group had a statistically significant greater number of CD45+ cells than the CAR 422 group. This indicates that the CAR M5 T cells continued to be activated and proliferate while failing to clear tumor while those mice that had cleared tumor had CAR T cells which have rested down presumably due to the lack of antigen. In the rechallenge, CD45+ cell levels were similar for the two stump-directed CARs. At the end of both studies, the vast majority of T cells in the ascites were CD4+ T cells.

CD45+ cells were also stained by a flow panel for CD62L and CD45RA to evaluate T cell memory phenotype (**Figure 5b**), for HLA-DR and TIM-3 to evaluate activation (**Figure 5c**), and for KLRG1/CD39 to evaluate T cell exhaustion (**Figure 5d**). The long-term CAR 422 and CAR 15B6 treatments had similar effector memory (EM) percentages which correlated to CAR M5 T cells in mice that controlled tumor. When tumor is uncontrolled, the CAR M5 mice have statistically more EM T cells while stump CAR T cells have statistically more central memory (CM) T cells after tumor clearance. The activation status of the T cells was then evaluated by staining for HLA-DR and TIM-3 expression (**Figure 5c**). In the long-term study, the CD45+ cells from stump-directed CARs 422 and 15B6, had an average HLA-DR+ expression of 35% and 40%, respectively, while the CAR M5 T cells showed a statistically significant higher level of activity, averaging around 75%. High levels of TIM-3 expression were detected only in the CAR M5 group. Comparing the CD45+ cells from the peritoneum of rechallenged stump-directed CARs, the percent HLA-DR+ expression on CAR 422 CD45+ cells was statistically lower relative to CAR 15B6. As tumor control was similar, and with HLA-DR being a late-stage activation marker used to distinguish activated from resting cells, this suggests CAR 422 may be less activated by soluble or membrane-bound MSLN stump and/or rest down faster than CAR 15B6. Minimal TIM-3 expression was expressed by either stump-directed CAR. Cells were stained for KLRG1 and CD39 surface expression to evaluate the exhaustion status of the persisting CD45+ T cells (**Figure 5d**) as dual expression is strongly correlated with exhaustion. In the long-term study, the percent of KLRG1+/CD39+ expression on CAR 422 CD45+ cells was statistically lower than that found on CAR M5 T cells. Negligible KLRG1+/CD39+ detection was found for the rechallenged CD45+ cells. Taken together, the stump-directed CARs show lower CD45+ cell numbers in the peritoneum after tumor clearance compared to CAR M5. Stump-directed CARs have an increased percentage of CM phenotype correlating with their absolute lower numbers and effective tumor control, are less activated, and do not appear to be exhausted when compared to CAR M5. These results also indicate that CAR 422 may be less activated than CAR 15B6 after tumor clearance, suggesting the possibility that CAR 422 may rest down more rapidly.

#### 2.3.3 Characterization of off-tumor toxicity

**Figure 6:**
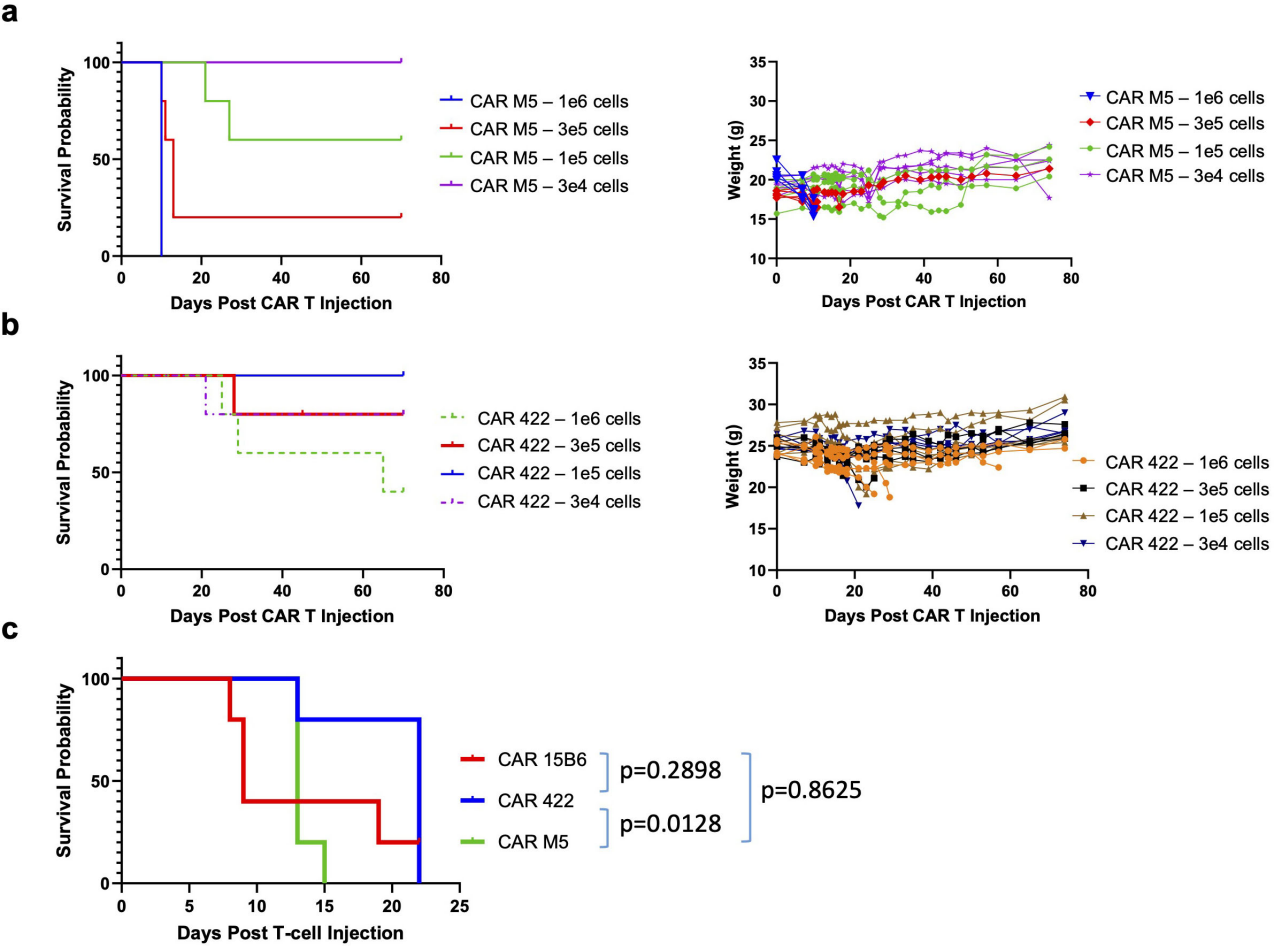
Evaluation of CAR T cell toxicity in huMSLN NSG mouse model. Non-tumor bearing huMSLN knock-in (KI) mice (n = 5 mice per group) were administered the indicated number of **(a)** CAR M5 or **(b)** CAR 422 T cells IV to assess off-tumor/on-target toxicity. Left-hand panels of **(a)** and **(b)** show probability of survival based on weight and health and right-hand panels of **(a)** and **(b)** show percent body weight change following T-cell injection as an indicator of toxicity and tolerability. **(c)** Non-tumor bearing huMSLN-KI mice (n=5 mice per group) were treated by injection of 1e6 CAR 422, 15B6, or M5 CAR T cells to evaluate relative toxicity. Statistical analysis used the Mantel-Cox log-rank test.

Given an enhanced efficacy of CAR 422 T cells targeting the MSLN stump domain in our OVCAR8 mouse model and their potential to have a lower degree of long-term activation, we wanted to compare off-tumor targeting responses of CAR 422 and CAR M5 to the endogenous MSLN expressed on normal tissue. For these studies, we used our established huMSLN-knock-in (KI) NSG tox mouse model in which huMSLN is expressed from the endogenous murine promoter by replacing mouse MSLN by insertional CRISPR technology (36). From previous studies, we have documented a highly reproducible dose titration curve of toxicity with our MSLN-directed CAR M5 T cells. **Figures 6a, b** compare the survival and weight of CAR M5 and CAR 422 T cell-treated mice at IV doses of 1e6, 3e5, 1e5 and 3e4 CAR+ T cells in groups of 5 huMSLN-KI mice. As we have observed previously for CAR M5 T cells at doses of 1e6, toxicity was detected in all mice on Day 10. Lower cell doses titrate down in terms of toxicity as measured by onset and severity of weight loss and ill health. Interestingly, for CAR 422 cells at the 1e6 dose, toxicity was not detected until Day 25 and only for a single mouse. On Day 29 a second mouse developed toxicity, but there was no further toxicity detected out to Day 45. The similarity between the degrees of toxicity between CAR 422 T cells at a dose of 1e6 and CAR M5+ T cells at 1e5 suggests a log decrease in dose toxicity for the CAR 422 T cells. In another study with different donor T cells, we compared CAR 442 with CAR 15B6 and M5 at a single IV dose of 1e6 CAR+ T cells which had shown strong differences in toxicity. **Figure 6c** again shows CAR 422 with a statistically significant reduced level of toxicity compared to CAR M5, whereas CAR 15B6 did not differ significantly from CAR M5. Survival following CARs 422 and 15B6 was also not statistically distinguishable, though CAR 422 yielded a higher fraction of long-term survivors. These observed reductions in off-tumor targeting of MSLN on normal tissue when combined with its MSLN stump-binding properties supports CAR 422 as a promising clinical candidate for treating MSLN shedding tumors with enhanced efficacy and safety.

### 2.4 Specificity of scFv ST4–22 for MSLN

Having identified CAR 422 as a lead candidate to advance into the clinic, we wanted to confirm monospecificity of the ST4–22 binder for MSLN. Rather than screen the ST4–22 antibody using conventional IHC-based tissue microarray technology with its documented limitations for predicting *in vivo* toxicity and safety (37, 38), ST4–22 off-target binding was assessed using the Integral Molecular Membrane Proteome Array™, a proprietary protein array comprising over 6,000 distinct human membrane protein clones each overexpressed in live cells from expression plasmids (39). The approach uses flow cytometry to directly detect antibody binding to human membrane proteins which includes 94% of all single-pass, multi-pass, and GPI-anchored proteins, GPCRs, ion channels, and transporters. As opposed to screening for off-target reactivity using formalin-fixed paraffin-embedded tissue as for 15B6 and R01 stump binders (22–24), proteins are expressed in unfixed cells in their native conformation with their appropriate post-translational modifications.

**Figure 7:**
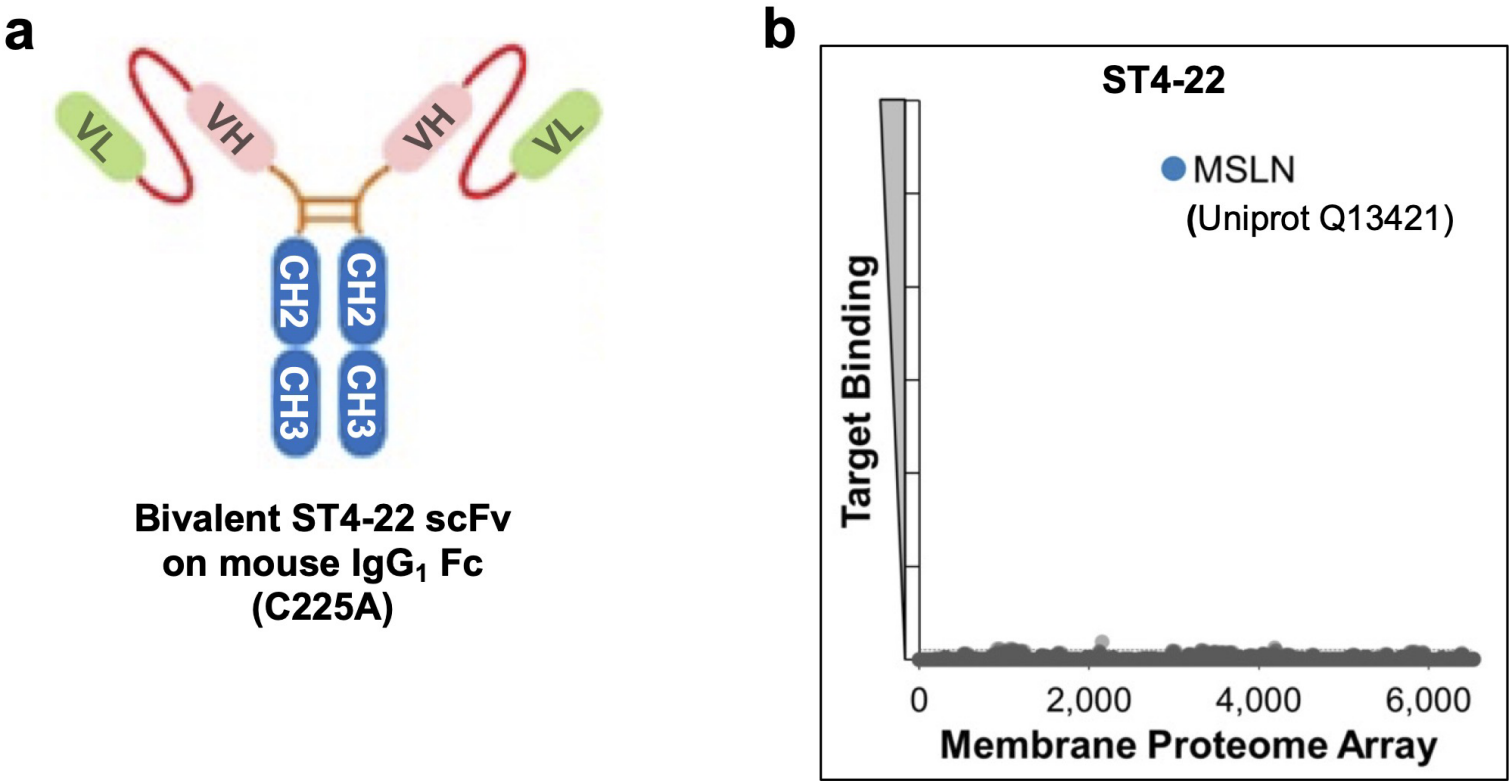
Specificity profiling of ST4–22 by testing cross-reactivity against a human membrane proteome array. **(a)** A bivalent scFv construct of ST4–22 was submitted to Integral Molecular, Inc. to probe their >6000-membrane human membrane proteome array. **(b)** Proteome screening demonstrated monospecificity for human mesothelin (Uniprot sequence Q13421).

To evaluate on the proteome array, ST4–22 was first expressed as a bivalent scFv using a murine Fc constant region (**Figure 7a**) and retention of binding to the appropriate form of MSLN was confirmed by ELISA (**Supplementary Figure 13**). To assess off-target binding, bivalent scFv ST4–22 was submitted to Integral Molecular for analysis. The results of the proteome array screening with ST4–22 are expressed graphically (**Figure 7b**) and indicated that ST4–22 has exquisite specificity for human MSLN (Uniprot Q13421).

## 3 Discussion

CAR T cells and other approaches targeting mesothelin have shown potential yet face challenges due to on-target/off-tumor toxicity and antigenic shedding (7, 26, 29, 40, 41). Development of CAR T cells targeting the juxtamembrane “stump” region of mesothelin such as CAR 422 T cells addresses long-standing challenges in CAR T cell therapy for solid tumors. Notably, shed mesothelin acts as both a decoy receptor and an immunosuppressive barrier binding CARs before they reach the tumor cell surface, reducing therapeutic efficacy (21, 22, 42). Our CAR 422 demonstrates comparable -- or superior -- cytotoxicity and *in vivo* tumor activity to conventional mesothelin-targeting CARs, owing to its resilience to shed antigen. In addition, CAR 422 T cells show marked reduction in off-tumor targeting of MSLN on normal tissues thus addressing a significant inherent safety concern with MSLN-directed CAR T cells tested in the clinic.

CAR 422’s ability to bind only the membrane-adjacent stump -- ignoring soluble, shed mesothelin -- mirrors findings with murine-derived 15B6 and its humanized h15B6 variant (22, 23). In patient-derived xenograft (PDX) models of pancreatic cancer, h15B6 CARs produced durable remissions where SS1-based CARs targeting the amino terminus of mesothelin failed (23). Our ST4–22 binder obtained using antibody phage display and a novel scFv library subtractive selection method further validates this approach, providing a scFv that avoids cross-competition with shed antigen and enables unimpeded tumor engagement.

Mesothelin shedding is a result of proteases from the tumor cells and those released from other cells in the tumor microenvironment which contributes to diminished antigen density on tumor cells, further reducing distal CAR efficacy (21). By binding the un-shed stump, CAR 422 effectively bypasses this barrier. Moreover, high concentrations of shed antigen in the tumor milieu have been associated with CAR T cell deactivation or sequestration at extraneous sites rather than the tumor (20, 22). Our construct avoids this trap, maximizing CAR T cell availability and function at the tumor interface while showing evidence of reduced off-target effects.

Human membrane proteome array screening confirmed that the scFv ST4–22 binder shows exquisite specificity for human mesothelin. Recent studies have confirmed that conventional approaches for assessing off-target reactivity including tissue cross-reactivity studies and tissue microarrays correlate poorly with clinical outcomes and *in vivo* patient safety (39, 43, 44). The proteome array assay employed in our studies is currently being evaluated by the FDA ISTAND (Innovative Science and Technology Approaches for New Drugs) program for full qualification as a drug development tool for assessing antibody specificity that is superior to conventional tissue-based methods (45). With respect to on-target/off-tumor toxicity, the use of huMSLN-KI mice offers a more appropriate toxicity model for evaluating huMSLN CAR T cells targeting normal tissues by expressing MSLN in a natural setting without the artifacts and potential issues associated with the laboratory processing of normal human tissue. A case in point was in the development of anti-MSLN M5 antibody where the M5 scFv was cloned into a full-length IgG antibody format and failed to effectively detect MSLN expression on fixed human tissues as opposed to commercially available anti-huMSLN IHC reagents which are specifically designed to detect MSLN in formalin-fixed, paraffin-embedded tissues (unpublished data). Thus, using huMSLN-KI mice allow us to directly compare the on-target/off-tumor toxicity of potential anti-MSLN stump CAR T cells with the clinically relevant M5 CAR and demonstrate an enhanced safety margin with CAR 422 in a physiological setting. Although CAR T cells utilizing scFvs 422 or 15B6 both engage the membrane-proximal MSLN stump, only CAR 422 demonstrated a statistically significant reduced lethality relative to the distal-targeting M5 CAR in this huMSLN-KI model. Stump membrane proximal epitope targeting therefore does not, on its own, account for the favorable safety profile of CAR 422. Though limitations in the use of the huMSLN-KI mouse model include unknown potential differences in MSLN shedding activity between mouse and human, we believe it is a useful tool to compare toxicities across MSLN-targeting CAR T cells independent of the relative activities of human and murine proteases. How much the observed enhanced safety of CAR 422 is due to intrinsic function of the specific CAR T cell or the precise MSLN stump epitope it targets has not yet been determined. It could be the case that there is a relative degree of steric occlusion for stump-directed CAR T cells due to the bulk of the MSLN membrane distal domains above the stump. In quiescent normal MSLN-expressing tissue where there is less activity of shedding enzymes (29), CAR 422 is not as active as a distal-binding CAR T cell such as CAR M5. It may also be the case that shed MSLN in normal tissues might transfer to bystander tissues that express MUC16/CA125 (e.g., peritoneum, pleura, lung) as suggested by Rump et al. (46) creating additional “ectopic” off-tumor, MSLN-specific, targets for membrane distal-binding CAR T cells but not CAR 422. Taken together, the results of membrane proteomic profiling of the ST-422 scFv and toxicity studies in the huMSLN-KI mice afford a superior safety profile for CAR T cell development than what is currently available for clinical development.

While high-affinity CAR T cells targeting MSLN have demonstrated potential off-tumor toxicity in preclinical lung models by performing “affinity tuning” of a single VHH nanobody (47), the precise role of affinity in efficacy and safety of an anti-MSLN CAR T cell is not at all clear. The relatively non-toxic SS1 anti-MSLN scFv has an affinity (K_D_ = 0.1 nM) that is more than 200-fold stronger than the M5 scFv (K_D_ = 26.9 nM) that resulted in pulmonary toxicity and patient fatalities (26). Affinity determination for ST-422 scFv has not been performed, but only Phase I clinical testing can fully evaluate the safety of CAR T cells targeting the stump epitope.

Although CAR 422 overcomes shedding-mediated resistance, broader challenges remain in solid-tumor CAR therapy: antigen heterogeneity, tumor infiltration, immunosuppressive microenvironment, T-cell exhaustion, and persistence (42, 48). Combining CAR 422 CAR T cells with checkpoint inhibitors, tumor microenvironment disruptors, “AND-gating” designs, engineered cytokine support, or by incorporation in a KIR-CAR signaling design (49), aligns with current strategies aimed at enhancing solid tumor efficacy. Future work could explore intratumoral or regional delivery routes to concentrate activity at tumor sites and limit systemic exposure, as has been tested with intrapleural administration in mesothelioma CAR T cell trials (6, 42, 50).

The activity of CAR 422 in tumors resistant to CARs targeting shed MSLN highlights its translational promise. Integration of this approach into early-phase clinical studies -- perhaps alongside tumor microenvironment disrupting agents -- could open new therapeutic opportunities in mesothelin-positive ovarian and pancreatic cancers. Drawing upon prior strategies with h15B6 constructs, advanced safety designs and combination regimens may optimize antitumor potency while maintaining safety (23).

In summary, ST4-22-expressing CAR T cells represent an advance in overcoming a key resistance mechanism in mesothelin-targeted immunotherapy as well as potentially reducing off-tumor toxicity. By precisely targeting the juxtamembrane stump, avoiding soluble antigen traps, maintaining specificity, and demonstrating reduced on-target/off-tumor toxicity in murine models expressing huMSLN, CAR 422 holds potential as a next-generation therapy for challenging solid tumors.

## 4 Materials and methods

### 4.1 Protein and immune reagents for scFv isolation and analysis

Streptavidin, neutravidin, and ABTS substrate were from Thermo Fisher Scientific (#43-4301, #31000, and #37615, respectively). Biotinylated MSLN (296-600), biotinylated MSLN (296-580), HIS-tag MSLN (296-580), and biotinylated LAIR1 were from ACROBiosystems (MSN-H82E7, MSN-H82E9, MDN-H522a, and LA1-H82E3, respectively). Anti-DNP murine IgG_1_ isotype control mAb was from ACROBiosystems (DNP-M1). HRP-conjugated anti-M13 murine mAb was from Sinobiologicals (11973-MM05T-H). HRP anti-mouse IgG, Fcγ fragment-specific was from Jackson Immunoresearch (115-035-071). Recombinant 15B6 (22), 30D12 anti-STEAP2 (51), and bivalent ST-422 were custom produced by Biointron Biological.

### 4.2 Phage display library panning

Antibody binding fragments specific to the stump region of MSLN were isolated from a canine scFv naïve/non-immune phage display library (31, 32) using a positive/negative solid phase panning strategy utilizing full-length MSLN (296-600) and MSLN (296-580) lacking the stump domain. To minimize disruption of the native conformations of MSLN proteins that can occur by direct adsorption to polystyrene substrates, MSLN proteins with a biotinylated AviTag™ were used and captured to streptavidin-coated 96-well microplates.

For each round of panning, a pair of 96-well Costar 3490 ½-area microplates were coated with streptavidin, 0.5 μg per well, at 4°C overnight (24 wells of each plate for panning round 1 and 8 wells of each plate for panning rounds 2 through 4). Plates were washed in PBS, and wells were blocked with 2% non-fat dry milk in PBS (MPBS) at 37°C for 1 h. To one plate in each pair, 36 pmol of biotinylated MSLN (296-600) was added to each streptavidin-coated well. To the other plate an equivalent amount of biotinylated MSLN (296-580) was added. After a 1-h incubation at 37°C, plates were washed with PBS to remove free MSLN proteins. Equal aliquots of μκ, μλ, γκ, and γλ scFv phage display libraries were combined, blocked for 1h at RT in 2% MPBS, and 50 μl added to each well of the MSLN (296-580)-coated plate. After a 1-h incubation at 37°C, negatively absorbed phage was applied to wells coated with MSLN (296-600) for positive selection on full-length MSLN. For panning rounds 2 through 4, negatively absorbed phage was additionally spiked with 1 nmol of non-biotinylated MSLN (296-580) before being added to the positive selection plate. Panning rounds and overnight phage amplification were continued as previously described (52, 53) where, after a 2-h incubation at 37°C, unbound phage were washed away 5 times during the first panning and 10 times during each subsequent panning round using PBS supplemented with 0.1% Tween 20 (PBST). Each wash was performed with a 5-min incubation of wash buffer in wells to select for binders with longer off-rates.

### 4.3 ScFv-phage ELISA

Monoclonal phage-displayed scFvs were prepared from panning eluates as described (54), added to microplates previously coated with streptavidin and biotinylated proteins and blocked with 2% MPBS as in panning procedure. From experience, a 1:100 dilution of overnight monoclonal scFv-phage-containing cultures provides consistent ELISA signals suitable for positive/negative screening. After washing away unbound phage with PBST, HRP-conjugated anti-M13 mAb (1:10,000) was added for 1h at 37°C and the microplate was washed, developed with ABTS substrate solution, and read at wavelengths of 410 nm and 490 nm.

### 4.4 15B6 competition ELISA

Microplate wells were coated with 0.5 μg/well streptavidin, blocked with 2% MPBS, and loaded with biotinylated MSLN (296-600). To increase the sensitivity of the assay, one-half of the amount of MSLN protein used in panning procedure was added per well (i.e., 18 pmol). For each scFv-phage to be tested, one well of a pair of MSLN-coated wells was left filled with blocking buffer and the other was filled with 15B6 diluted in blocking buffer. A titration of 15B6 (**Supplementary Figure 2**) had indicated that under these conditions, 15B6 binding saturated at ~1 μg/mL. Therefore, a 10-fold excess of 15B6 (10 μg/mL = 0.13 μM/L of monovalent equivalent of IgG) was used here. Microplates were incubated for 30 min at 37°C to allow 15B6 to bind and then 1/9^th^ volume of 10x-strength scFv-phage was added to each well of a pair to bring the final scFv-phage concentration to standard ELISA phage dilution of 1:100 (~2.5e8 phage/ml = 0.42 pM/L). After an additional 1h at 37°C, wells were washed with PBST, developed with HRP-conjugated anti-M13, and developed with ABTS substrate as above. At this molar ratio of 15B6 IgG over scFv (~300,000:1), the binding of scFv with higher affinity than 15B6 would still be blocked by 15B6.

### 4.5 Nucleotide sequencing of scFv clones and analysis

Mini-scale DNA plasmid preparations of the pComb3X phagemid vector were made from monoclonal scFv-phage cultures, and nucleotide sequences for the heavy and light chain scFv variable regions were determined using the pComb3X vector-specific sequencing primers “5’LC” and “dpseq” that anneal just upstream of the light chain and downstream of the heavy chain variable region sequences, respectively (30). Sequencing was performed by the University of Pennsylvania Department of Genetics DNA Sequencing Core Facility using the di-deoxynucleotide chain termination (Sanger) method on an ABI 3730xl Sequencer.

Nucleotide sequences were analyzed using MacVector software (v. 18.7.8). The online IMGT/V-QUEST germline gene analysis tool (55) available at https://www.imgt.org/IMGT_vquest/input and the online IMGT/DomainGapAlign immunoglobulin protein analysis tool (56) available at https://www.imgt.org/3Dstructure-DB/cgi/DomainGapAlign.cgi were used together to identify V, D, and J germline genes and identify framework regions and CDRs.

### 4.6 Membrane proteome array screening

Bivalent scFv ST4–22 was analyzed on the Integral Molecular Membrane Proteome Array™ (39). Each of over 6,000 distinct membrane protein clones was individually transfected in separate wells of 384-well plates followed by a 24-h incubation. Cells expressing each individual protein clone were arrayed in duplicate in a matrix format for high-throughput screening. Before screening on the array, the ST-422 concentration for screening was determined on cells expressing positive (membrane-tethered Protein A) and negative (mock- transfected) binding controls, followed by detection by flow cytometry using a fluorescently labeled secondary antibody. ST-422 was added to the array at the predetermined concentration, and binding across the protein library was measured on an Intellicyt iQue using an AlexaFluorR 647 labeled goat F(ab’)_2_ anti-mouse Fc secondary antibody (Jackson ImmunoResearch 115-606-008). Each array plate contained both positive (Fc-binding) and negative (empty vector) controls to ensure plate-to-plate reproducibility. Test ligand interactions with any targets identified by membrane protein array screening were confirmed in a second flow cytometry experiment using serial dilutions of the test antibody, and the target identity was re-verified by sequencing.

### 4.7 Lentiviral vector construction

All scFvs evaluated in our pTRPE lentiviral CAR format were synthesized (GenScript) and cloned after the CD8α leader sequence and fused to CD8α hinge and transmembrane domains and the intracellular signal domains of 4-1BB and CD3ς. The CAR construct for scFv ST-422 was evaluated for killing of AsPC1 cells using both our conventional 20-mer linker [4x(G_4_S)] as well as 18-mer standard linker used in the design of pComb3X scFv phage display libraries (30) (**Supplementary Figure 14**). The construct used for CAR 15B6 used the 15-mer [3x(G_4_S)] as described (22). For construct expression of carboxy 17 aa stump, a transgene encoding the MSLN leader sequence, 3x FLAG tag, the 17aa stump along with the huMSLN carboxy GPI signaling domain were synthesized (GenScript) downstream of EF1α promoter driving eGFP expression and T2A expression.

### 4.8 Engineering human CAR T cells

Lentiviral constructs encoding CAR transgenes were packaged, titered on SupT1 cells, and transduced into human T-cells activated by CD3/CD28-Dynabeads (Thermo Fisher Scientific, #43500D) as previously described (57). Transduced T cells were cultured and expanded in X-VIVO 15 (Lonza Biosciences, #02-060Q) media supplemented with 5% HuAB serum (BioIVT, #HUMANABSRMP-HI-1) and supplemented with 5 ng/mL IL-7 and IL-15 (Miltenyi Biotec Inc., #130-095–363 and #130-095-765, respectively) until cell volumes reduced to around 350 fL before freezing. CAR transduction efficiencies were determined from surface expression by flow cytometry staining for human M5 and 119 scFv with biotinylated goat anti-human IgG F(ab’)_2_ fragment antibody (Jackson ImmunoResearch, #109-066-006), for murine 15B6 with biotinylated goat anti-mouse IgG F(ab’)_2_ fragment antibody (Jackson ImmunoResearch, #115-065-072), and for canine phage library-derived CARs with biotinylated rabbit anti-dog IgG F(ab’)_2_ fragment antibody (Rockland, #604-4604), all followed by secondary staining with phycoerythrin-conjugated streptavidin (BioLegend, #405203).

### 4.9 Tumor cell lines

AsPC1, a pancreatic adenocarcinoma cell line (ATCC CRL-1682), H838, a non-small cell lung adenocarcinoma (ATCC CRL-5844), and OVCAR8, an ovarian adenocarcinoma (RRID: CVCL-1629) were each transduced to express eGFP-2A-CBR (click beetle red) for monitoring MSLN CAR targeting. All cells were authenticated by autosomal DNA profiling by the University of Arizona, Tucson, AZ. MSLN expression was knocked out in AsPC1 and H838 cells using CRISPR technology for evaluating non-MSLN activity of CAR T cells. AsPC1 and H838 MSLN KO cells were lentivirus transduced to express only the carboxy 17 aa stump region with transduction confirmed by GFP and expression by a FLAG tag. Additional cell lines used for soluble MSLN studies included KLM-1, a human pancreatic adenocarcinoma cell line obtained through a material transfer agreement with Riken BioResource Center, Tsukuba, Japan, and SK-OV-3 cells and OVCAR3 cells, both human ovarian adenocarcinoma cell line obtained from ATCC (SKOV-3 and OVCAR3, respectively).

### 4.10 Incucyte^®^ assays

eGFP transduced tumor cells (AsPC1, H838, OVCAR8) were cocultured with CAR^+^ T cells at various E:T ratios. Signal intensities were quantified every 3 hours for 7 days. Incucyte^®^ Cytotox NIR Dye was added at 1:200 dilution to each well to monitor cell death. CAR killing activity was quantified by decrease in GFP fluorescent intensity using Incuyte^®^ SX5’s Adherent Cell-by-Cell analysis software. The percent cell killing was calculated as {[1- (GFP intensity of treatment/GFP intensity of control)] * 100}.

### 4.11 Chronic antigen exposure stress test

GFP positive AsPC1 cells were co-cultured with CAR T cells at a 1:1 E:T ratio. Incucyte^®^ Cytotox Dye for Counting Dead Cells was added at 1:200 dilution to each well. CAR killing activity was monitored by the Incucyte^®^ every 3 hours. CAR T cells were restimulated with new AsPC1 cells when 50% killing was achieved. At restimulation, the media and T cells were collected and centrifuged. Fresh AsPC1 cells were re-seeded onto the plate. Half of the conditioned media and all of the T cells were added back to the well. Restimulation was repeated until the exhaustion profile was characterized.

### 4.12 Chronic antigen exposure flow analysis

At the end of each restimulation, cells were removed to determine the CAR T cell number and their phenotype by flow cytometric analysis. Cells were evaluated for viability with LIVE/DEAD™ Fixable Aqua Dead Cell Stain Kit (Thermo Fisher Scientific, #L34966) then stained for surface CAR expression with Biotin-SP AffiniPure^®^ F(ab’)_2_ Fragment Goat Anti-Human IgG (Jackson ImmunoResearch, #109-066-006), Biotin-SP AffiniPure^®^ Goat Anti-Mouse IgG (Jackson ImmunoResearch, #115-065-072), or Rabbit Anti-Dog IgG F(ab’)_2_ Biotin Conjugated Affinity purified (Rockland, #604-4604), followed by PE-Streptavidin (BioLegend, #405203) secondary staining. The following human markers were measured: CD45 (BioLegend, #304011), CD4 (BioLegend, #317442), CD8 (BioLegend, #3009100), PD-1 (BioLegend, #329928), TIM-3 (BioLegend, #345018), TIGIT (BioLegend, #372714), KLRG1 (BioLegend, #368604). CountBright Absolute Counting Beads (Invitrogen, #C36950) were used to quantify cell numbers. Samples were run on a BD LSR Fortessa and data analyzed with FlowJo v10.10.0 software. Supernatants evaluated for levels of cytokines using the Human Cytokine Proinflammatory Focused 15-Plex Discovery Assay^®^ Array (HDF15, Eve Technologies, Alberta CN).

### 4.13 Mouse models

NOD *scid* gamma mice were supplied by the Stem Cell and Xenograft Core of the University of Pennsylvania. For the AsPC1 mouse model, 2e6 AsPC1 cells suspended in 50% Matrigel (Corning, #356234) were injected into the flank subcutaneously. Mice were treated with CAR T cells when average of tumor volumes reached ~150 mm^3^. Tumor sizes were measured weekly and weights measured biweekly.

For the OVCAR8 NSG mouse model, 3e6 OVCAR8 cells were injected into the peritoneal cavity and allowed to grow for about 3 weeks before CAR T cell IV injection. Tumor progression was measured weekly by bioluminescence using the IVIS Lumina system, and data was analyzed using Living Image^®^. Weights were measured biweekly. Pre-specified endpoints of animal experiments were the animals’ health and tumor burden. All procedures followed the guidelines of an IACUC-approved animal protocol at the University of Pennsylvania.

HuMSLN-KI NSG mice were generated by contract to Jackson Labs Services (36) (manuscript in preparation).

### 4.14 Peritoneum and ascites collection and analysis

Peritoneal fluid without ascites was collected by first injecting 5 mL of 3% FBS buffer with a 27-G syringe into the peritoneal cavity, followed by periodic massaging of belly over a 10-min period, and fluid collection using 25-G syringes. Ascites fluid collection was performed directly using 25-G syringes. The collected cells were analyzed by flow cytometry for the number of CD45+ T cell numbers and their phenotypes. Soluble human mesothelin was quantified by ELISA (ThermoFisher, #EH322RB). Red blood cells were removed from ascites with ACK RBC lysis buffer (Lonza Biosciences, #BP10-548E). The resulting peritoneal and ascites cells were stained for the following human markers: CD45 (BioLegend, #368516), CD4 (BioLegend, #317434 or #317434), CD8 (BioLegend, #344742), CD45RO (BioLegend, #304210), CD62L (BioLegend, #304822), HLA-DR (BioLegend, #327022), TIM-3 (BioLegend, #345006), PD-1 (BioLegend, #329928) and CD39 (BioLegend, #328224). Mouse myeloid markers evaluated were CD45 (BioLegend, #368516) and CD11b (BioLegend, #550993).

## Data Availability

The original contributions presented in the study are included in the article/**Supplementary Material**, further inquiries can be directed to the corresponding authors.
