# Supplementary material for: Engineering a juxtamembrane-targeting CAR T-cell against mesothelin: a novel binder resilient to shed antigen for enhanced efficacy against ovarian and pancreatic cancer
Source: Front Immunol. 2026 Jul 1;17:1805950. doi: 10.3389/fimmu.2026.1805950 (PMC13369438; doi:10.3389/fimmu.2026.1805950)
Supplement: Supplementary file 1 [file DataSheet1.pdf]

Supplementary Table 1. Amino acid sequences of MSLN stump-binding scFvs

CLONE	scFv (LC - linker - HC) where linker =GGSSRSSSSGGGGSGGGG	LIGHT CHAIN	HEAVY CHAIN	HC CDR1	HC CDR2	HC CDR3	LC CDR1	LC CDR2	LC CDR3
**P3-09**	DIVMTQTPLSLSVSPGEPASISCKASQSLLHSDGNTYLYWFRQKPGQSPQRLIYLVSNRDAGVPDRFSGSGSGTDFTLRINRVEANDSGSYYCGQGLQYPITFGEGTHLEIKGGSSRSSSSGGGGSGGGGELTLQESGPGLVKPSQTLSLTCVVSGGSVTSNYYWHWIRQRPGRGLEWMGSWAASTNYNPAFQGRISITADTAKNQFSLQLTSMTTEDTAVYYCARNSGSWGQGTLVTVSS	DIVMTQTPLSLSVSPGEPASISCKASQSLLHSDGNTYLYWFRQKPGQSPQRLIYLVSNRDAGVPDRFSGSGSGTDFTLRINRVEANDSGSYYCGQGLQYPITFGEGTHLEIK	ELTLQESGPGLVKPSQTLSLTCVVSGGSVTSNYYWHWIRQRPGRGLEWMGSWAASTNYNPAFQGRISITADTAKNQFSLQLTSMTTEDTAVYYCARNSGSWGQGTLVTVSS	GGSVTSNYY	WAAST	ARNSGS	QSLLHSDGNTY	LVS	GQGLQYPIT
**P3-14**	DIVMTQNPLSLSVSPGEPASISCKASQSLLHSNGNTYLYWFRQKPGQSPQRLIYLLSNRDAGVPDRFSGSGSGTDFTLRISRVEADDTGVYYCGQGIQYPVTFGQGTKLEIKGGSSRSSSSGGGGSGGGGELTLQESGPGLVKPSQTLSLTCVVSGGSVTSNYYWHWIRQRPGRGLEWMGSWAASTNYNPAFQGRISITADTAKNQFSLQLTSMTTEDTAVYYCARNSGSWGQGTLVTVSS	DIVMTQNPLSLSVSPGEPASISCKASQSLLHSNGNTYLYWFRQKPGQSPQRLIYLLSNRDAGVPDRFSGSGSGTDFTLRISRVEADDTGVYYCGQGIQYPVTFGQGTKLEIK	ELTLQESGPGLVKPSQTLSLTCVVSGGSVTSNYYWHWIRQRPGRGLEWMGSWAASTNYNPAFQGRISITADTAKNQFSLQLTSMTTEDTAVYYCARNSGSWGQGTLVTVSS	GGSVTSNYY	WAAST	ARNSGS	QSLLHSNGNTY	LLS	GQGIQYPVT
**P4-11**	EAVMTQTPLSLAVTPGEVVTISCRASQSLLRSSDGKSYLNWYLQKPGQTPRPLIYEASKRFSGVSDRFSGSGSGTDFTLKISRVEAEDVGVYYCQQSLHFPPTFGAGTKVELKGGSSRSSSSGGGGSGGGGEVQLVESGGDLVKPGGSLRLSCVASGFSFSTYGMSWVRQSPGKGLQWVASIRRDGSDTYYTDAVEGRFTISRDNANNTLYLQMNSLRAEDTAVYYCARDGNNWSWDYWGHGTSLFVPL	EAVMTQTPLSLAVTPGEVVTISCRASQSLLRSSDGKSYLNWYLQKPGQTPRPLIYEASKRFSGVSDRFSGSGSGTDFTLKISRVEAEDVGVYYCQQSLHFPPTFGAGTKVELK	EVQLVESGGDLVKPGGSLRLSCVASGFSFSTYGMSWVRQSPGKGLQWVASIRRDGSDTYYTDAVEGRFTISRDNANNTLYLQMNSLRAEDTAVYYCARDGNNWSWDYWGHGTSLFVPL	GFSFSTYG	IRRDGSDT	ARDGNNWSWDY	QSLLRSSDGKSY	EAS	QQSLHFPPT
**P4-22**	EAVMTQTPLSLAVTPGELATISCRASQSLLHRDGKSYLSWYLQKPGQAPRPLIYEASKRFSGVSDRFSGSGSGTDFTLKISRVEAEDVGVYYCQQSLHFPPTFGQGTKVEIKGGSSRSSSSGGGGSGGGGEVPLVESGGDLVKPGGSLRLSCISSGFTFSDYGMTWVRQSPGKGLQWVASIRRDGSDTYYTDAVEGRFTISRDNANNTLYLQMNSLRAEDTAVYYCARDGNNWSWDYWGHGTSLFVSS	EAVMTQTPLSLAVTPGELATISCRASQSLLHRDGKSYLSWYLQKPGQAPRPLIYEASKRFSGVSDRFSGSGSGTDFTLKISRVEAEDVGVYYCQQSLHFPPTFGQGTKVEIK	EVPLVESGGDLVKPGGSLRLSCISSGFTFSDYGMTWVRQSPGKGLQWVASIRRDGSDTYYTDAVEGRFTISRDNANNTLYLQMNSLRAEDTAVYYCARDGNNWSWDYWGHGTSLFVSS	GFTFSDYG	IRRDGSDT	ARDGNNWSWDY	QSLLHRDGKSY	EAS	QQSLHFPPT
**P4-27**	DIVMTQTPLSLSVSPGEPASISCKASQSLLHSDGNTYLYWFRQKAGQSPQRLISLVSKRDAGVPDRVSGSGSGTDFTLRISRVEADDAGIYYCGQGLQYPITFGQGTKVEIKGGSSRSSSSGGGGSGGGGELTLQESGPGLVKPSQTLSLTCVVSGGSVTSNYYWHWIRRRPGRGLEWMGSWAASTNYNPAFQGRISITADTAKNQFSLQLTSMTTEDTAVYYCARNSGSWGQGTLVTVSS	DIVMTQTPLSLSVSPGEPASISCKASQSLLHSDGNTYLYWFRQKAGQSPQRLISLVSKRDAGVPDRVSGSGSGTDFTLRISRVEADDAGIYYCGQGLQYPITFGQGTKVEIK	ELTLQESGPGLVKPSQTLSLTCVVSGGSVTSNYYWHWIRRRPGRGLEWMGSWAASTNYNPAFQGRISITADTAKNQFSLQLTSMTTEDTAVYYCARNSGSWGQGTLVTVSS	GGSVTSNYY	WAAST	ARNSGS	QSLLHSDGNTY	LVS	GQGLQYPIT
**P4-39**	EAVMTQTPLSLSVSPGETASISCKASQNLLHSNGNTYLNWFRQKPGQSPEGLIYEVSNRVPGVPDRFSGSGSGTDFTLRISRVEADDAGVYYCGQATQAPITFGKGTHLEIKGGSSRSSSSGGGGSGGGGEVQLVESGGDLVKPGGSLRLSCVASGFTFSNYGMNWVRQAPGKELQWVAAISYDGSNTYYTDDVKGRFTISRDNAKNTVNLQMNSLRAEDTAMYYCVKGGYFDYWGRGTLVTVSS	EAVMTQTPLSLSVSPGETASISCKASQNLLHSNGNTYLNWFRQKPGQSPEGLIYEVSNRVPGVPDRFSGSGSGTDFTLRISRVEADDAGVYYCGQATQAPITFGKGTHLEIK	EVQLVESGGDLVKPGGSLRLSCVASGFTFSNYGMNWVRQAPGKELQWVAAISYDGSNTYYTDDVKGRFTISRDNAKNTVNLQMNSLRAEDTAMYYCVKGGYFDYWGRGTLVTVSS	GFTFSNYG	ISYDGSNT	VKGGYFDY	QNLLHSNGNTY	EVS	GQATQAPIT

Supplementary Table 2. Nucleotide sequences for vector before (ST-422) and after (CAR 422) ORF optimization

ST-422	CAR 422
GTGCACGAGTGGGTTACATCGAACTGGATCTCAACAGCGGTAAGATCCTTGAGAGTTTTCGCC	GTGCACGAGTGGGTTACATCGAACTGGATCTCAACAGCGGTAAGATCCTTGAGAGTTTTCGCC
CCGAAGAACGTTTTCCAATGATGAGCACTTTTAAAGTTCTGCTATGTGGCGCGGTATTATCCC	CCGAAGAACGTTTTCCAATGATGAGCACTTTTAAAGTTCTGCTATGTGGCGCGGTATTATCCC
GTATTGACGCCGGGCAAGAGCAACTCGGTCGCCGCATACACTATTCTCAGAATGACTTGGTTG	GTATTGACGCCGGGCAAGAGCAACTCGGTCGCCGCATACACTATTCTCAGAATGACTTGGTTG
AGTACTCACCAGTCACAGAAAAGCATCTTACGGATGGCATGACAGTAAGAGAATTATGCAGTG	AGTACTCACCAGTCACAGAAAAGCATCTTACGGATGGCATGACAGTAAGAGAATTATGCAGTG
CTGCCATAACCATGAGTGATAACACTGCGGCCAACTTACTTCTGACAACGATCGGAGGACCGA	CTGCCATAACCATGAGTGATAACACTGCGGCCAACTTACTTCTGACAACGATCGGAGGACCGA
AGGAGCTAACCGCTTTTTTGCACAACATGGGGGATCATGTAACTCGCCTTGATCGTTGGGAAC	AGGAGCTAACCGCTTTTTTGCACAACATGGGGGATCATGTAACTCGCCTTGATCGTTGGGAAC
CGGAGCTGAATGAAGCCATACCAAACGACGAGCGTGACACCACGATGCCTGTAGCAATGGCAA	CGGAGCTGAATGAAGCCATACCAAACGACGAGCGTGACACCACGATGCCTGTAGCAATGGCAA
CAACGTTGCGCAAACTATTAACTGGCGAACTACTTACTCTAGCTTCCCGGCAACAATTAATAG	CAACGTTGCGCAAACTATTAACTGGCGAACTACTTACTCTAGCTTCCCGGCAACAATTAATAG
ACTGGATGGAGGCGGATAAAGTTGCAGGACCACTTCTGCGCTCGGCCCTTCCGGCTGGCTGGT	ACTGGATGGAGGCGGATAAAGTTGCAGGACCACTTCTGCGCTCGGCCCTTCCGGCTGGCTGGT
TTATTGCTGATAAATCTGGAGCCGGTGAGCGTGGGTCTCGCGGTATCATTGCAGCACTGGGGC	TTATTGCTGATAAATCTGGAGCCGGTGAGCGTGGGTCTCGCGGTATCATTGCAGCACTGGGGC
CAGATGGTAAGCCCTCCCGTATCGTAGTTATCTACACGACGGGGAGTCAGGCAACTATGGATG	CAGATGGTAAGCCCTCCCGTATCGTAGTTATCTACACGACGGGGAGTCAGGCAACTATGGATG
AACGAAATAGACAGATCGCTGAGATAGGTGCCTCACTGATTAAGCATTGGTAACTGTCAGACC	AACGAAATAGACAGATCGCTGAGATAGGTGCCTCACTGATTAAGCATTGGTAACTGTCAGACC
AAGTTTACTCATATATACTTTAGATTGATTTAAAACTTCATTTTTAATTTAAAAGGATCTAGG	AAGTTTACTCATATATACTTTAGATTGATTTAAAACTTCATTTTTAATTTAAAAGGATCTAGG
TGAAGATCCTTTTTGATAATCTCATGACCAAAATCCCTTAACGTGAGTTTTCGTTCCACTGAG	TGAAGATCCTTTTTGATAATCTCATGACCAAAATCCCTTAACGTGAGTTTTCGTTCCACTGAG
CGTCAGACCCCGTAGAAAAGATCAAAGGATCTTCTTGAGATCCTTTTTTTCTGCGCGTAATCT	CGTCAGACCCCGTAGAAAAGATCAAAGGATCTTCTTGAGATCCTTTTTTTCTGCGCGTAATCT
GCTGCTTGCAAACAAAAAAACCACCGCTACCAGCGGTGGTTTGTTTGCCGGATCAAGAGCTAC	GCTGCTTGCAAACAAAAAAACCACCGCTACCAGCGGTGGTTTGTTTGCCGGATCAAGAGCTAC
CAACTCTTTTTCCGAAGGTAACTGGCTTCAGCAGAGCGCAGATACCAAATACTGTTCTTCTAG	CAACTCTTTTTCCGAAGGTAACTGGCTTCAGCAGAGCGCAGATACCAAATACTGTTCTTCTAG
TGTAGCCGTAGTTAGGCCACCACTTCAAGAACTCTGTAGCACCGCCTACATACCTCGCTCTGC	TGTAGCCGTAGTTAGGCCACCACTTCAAGAACTCTGTAGCACCGCCTACATACCTCGCTCTGC
TAATCCTGTTACCAGTGGCTGCTGCCAGTGGCGATAAGTCGTGTCTTACCGGGTTGGACTCAA	TAATCCTGTTACCAGTGGCTGCTGCCAGTGGCGATAAGTCGTGTCTTACCGGGTTGGACTCAA
GACGATAGTTACCGGATAAGGCGCAGCGGTCGGGCTGAACGGGGGGTTCGTGCACACAGCCCA	GACGATAGTTACCGGATAAGGCGCAGCGGTCGGGCTGAACGGGGGGTTCGTGCACACAGCCCA
GCTTGGAGCGAACGACCTACACCGAACTGAGATACCTACAGCGTGAGCTATGAGAAAGCGCCA	GCTTGGAGCGAACGACCTACACCGAACTGAGATACCTACAGCGTGAGCTATGAGAAAGCGCCA
CGCTTCCCGAAGGGAGAAAGGCGGACAGGTATCCGGTAAGCGGCAGGGTCGGAACAGGAGAGC	CGCTTCCCGAAGGGAGAAAGGCGGACAGGTATCCGGTAAGCGGCAGGGTCGGAACAGGAGAGC
GCACGAGGGAGCTTCCAGGGGGAAACGCCTGGTATCTTTATAGTCCTGTCGGGTTTCGCCACC	GCACGAGGGAGCTTCCAGGGGGAAACGCCTGGTATCTTTATAGTCCTGTCGGGTTTCGCCACC
TCTGACTTGAGCGTCGATTTTTGTGATGCTCGTCAGGGGGGCGGAGCCTATGGAAAAACGCCA	TCTGACTTGAGCGTCGATTTTTGTGATGCTCGTCAGGGGGGCGGAGCCTATGGAAAAACGCCA
GCAACGCGGCCTTTTTACGGTTCCTGGCCTTTTGCTGGCCTTTTGCTCACATGTTCTTTCCTG	GCAACGCGGCCTTTTTACGGTTCCTGGCCTTTTGCTGGCCTTTTGCTCACATGTTCTTTCCTG
CGTTATCCCCTGATTCTGTGGATAACCGTATTACCGCCTTTGAGTGAGCTGATACCGCTCGCC	CGTTATCCCCTGATTCTGTGGATAACCGTATTACCGCCTTTGAGTGAGCTGATACCGCTCGCC
GCAGCCGAACGACCGAGCGCAGCGAGTCAGTGAGCGAGGAAGCGGAAGAGCGCCCAATACGCA	GCAGCCGAACGACCGAGCGCAGCGAGTCAGTGAGCGAGGAAGCGGAAGAGCGCCCAATACGCA
AACCGCCTCTCCCCGCGCGTTGGCCGATTCATTAATGCAGCTGGCACGACAGGTTTCCCGACT	AACCGCCTCTCCCCGCGCGTTGGCCGATTCATTAATGCAGCTGGCACGACAGGTTTCCCGACT
GGAAAGCGGGCAGTGAGCGCAACGCAATTAATGTGAGTTAGCTCACTCATTAGGCACCCCAGG	GGAAAGCGGGCAGTGAGCGCAACGCAATTAATGTGAGTTAGCTCACTCATTAGGCACCCCAGG
CTTTACACTTTATGCTTCCGGCTCGTATGTTGTGTGGAATTGTGAGCGGATAACAATTTCACA	CTTTACACTTTATGCTTCCGGCTCGTATGTTGTGTGGAATTGTGAGCGGATAACAATTTCACA
CAGGAAACAGCTATGACCATGATTACGCCAAGCGCGCAATTAACCCTCACTAAAGGGAACAAA	CAGGAAACAGCTATGACCATGATTACGCCAAGCGCGCAATTAACCCTCACTAAAGGGAACAAA
AGCTGGAGCTGCAAGCTTAATGTAGTCTTATGCAATACTCTTGTAGTCTTGCAACATGGTAAC	AGCTGGAGCTGCAAGCTTAATGTAGTCTTATGCAATACTCTTGTAGTCTTGCAACATGGTAAC
GATGAGTTAGCAACATGCCTTACAAGGAGAGAAAAAGCACCGTGCATGCCGATTGGTGGAAGT	GATGAGTTAGCAACATGCCTTACAAGGAGAGAAAAAGCACCGTGCATGCCGATTGGTGGAAGT
AAGGTGGTACGATCGTGCCTTATTAGGAAGGCAACAGACGGGTCTGACATGGATTGGACGAAC	AAGGTGGTACGATCGTGCCTTATTAGGAAGGCAACAGACGGGTCTGACATGGATTGGACGAAC
CACTGAATTGCCGCATTGCAGAGATATTGTATTTAAGTGCCTAGCTCGATACATAAACGGGTC	CACTGAATTGCCGCATTGCAGAGATATTGTATTTAAGTGCCTAGCTCGATACATAAACGGGTC
TCTCTGGTTAGACCAGATCTGAGCCTGGGAGCTCTCTGGCTAACTAGGGAACCCACTGCTTAA	TCTCTGGTTAGACCAGATCTGAGCCTGGGAGCTCTCTGGCTAACTAGGGAACCCACTGCTTAA
GCCTCAATAAAGCTTGCCTTGAGTGCTTCAAGTAGTGTGTGCCCGTCTGTTGTGTGACTCTGG	GCCTCAATAAAGCTTGCCTTGAGTGCTTCAAGTAGTGTGTGCCCGTCTGTTGTGTGACTCTGG
TAACTAGAGATCCCTCAGACCCTTTTAGTCAGTGTGGAAAATCTCTAGCAGTGGCGCCCGAAC	TAACTAGAGATCCCTCAGACCCTTTTAGTCAGTGTGGAAAATCTCTAGCAGTGGCGCCCGAAC
AGGGACTTGAAAGCGAAAGGGAAACCAGAGGAGCTCTCTCGACGCAGGACTCGGCTTGCTGAA	AGGGACTTGAAAGCGAAAGGGAAACCAGAGGAGCTCTCTCGACGCAGGACTCGGCTTGCTGAA
GCGCGCACGGCAAGAGGCGAGGGGCGGCGACTGGTGAGTACGCCAAAAATTTTGACTAGCGGA	GCGCGCACGGCAAGAGGCGAGGGGCGGCGACTGGTGAGTACGCCAAAAATTTTGACTAGCGGA
GGCTAGAAGGAGAGAGATGGGTGCGAGAGCGTCAGTATTAAGCGGGGGAGAATTAGATCGCGA	GGCTAGAAGGAGAGAGATGGGTGCGAGAGCGTCAGTATTAAGCGGGGGAGAATTAGATCGCGA
TGGGAAAAAATTCGGTTAAGGCCAGGGGGAAAGAAAAAATATAAATTAAAACATATAGTATGG	TGGGAAAAAATTCGGTTAAGGCCAGGGGGAAAGAAAAAATATAAATTAAAACATATAGTATGG
GCAAGCAGGGAGCTAGAACGATTCGCAGTTAATCCTGGCCTGTTAGAAACATCAGAAGGCTGT	GCAAGCAGGGAGCTAGAACGATTCGCAGTTAATCCTGGCCTGTTAGAAACATCAGAAGGCTGT
AGACAAATACTGGGACAGCTACAACCATCCCTTCAGACAGGATCAGAAGAACTTAGATCATTA	AGACAAATACTGGGACAGCTACAACCATCCCTTCAGACAGGATCAGAAGAACTTAGATCATTA
TATAATACAGTAGCAACCCTCTATTGTGTGCATCAAAGGATAGAGATAAAAGACACCAAGGAA	TATAATACAGTAGCAACCCTCTATTGTGTGCATCAAAGGATAGAGATAAAAGACACCAAGGAA
GCTTTAGACAAGATAGAGGAAGAGCAAAACAAAAGTAAGACCACCGCACAGCAAGCGGCCGCT	GCTTTAGACAAGATAGAGGAAGAGCAAAACAAAAGTAAGACCACCGCACAGCAAGCGGCCGCT
GATCTTCAGACCTGGAGGAGGAGATATGAGGGACAATTGGAGAAGTGAATTATATAAATATAA	GATCTTCAGACCTGGAGGAGGAGATATGAGGGACAATTGGAGAAGTGAATTATATAAATATAA
AGTAGTAAAAATTGAACCATTAGGAGTAGCACCCACCAAGGCAAAGAGAAGAGTGGTGCAGAG	AGTAGTAAAAATTGAACCATTAGGAGTAGCACCCACCAAGGCAAAGAGAAGAGTGGTGCAGAG
AGAAAAAAGAGCAGTGGGAATAGGAGCTTTGTTCCTTGGGTTCTTGGGAGCAGCAGGAAGCAC	AGAAAAAAGAGCAGTGGGAATAGGAGCTTTGTTCCTTGGGTTCTTGGGAGCAGCAGGAAGCAC
TATGGGCGCAGCGTCAATGACGCTGACGGTACAGGCCAGACAATTATTGTCTGGTATAGTGCA	TATGGGCGCAGCGTCAATGACGCTGACGGTACAGGCCAGACAATTATTGTCTGGTATAGTGCA
GCAGCAGAACAATTTGCTGAGGGCTATTGAGGCGCAACAGCATCTGTTGCAACTCACAGTCTG	GCAGCAGAACAATTTGCTGAGGGCTATTGAGGCGCAACAGCATCTGTTGCAACTCACAGTCTG
GGGCATCAAGCAGCTCCAGGCAAGAATCCTGGCTGTGGAAAGATACCTAAAGGATCAACAGCT	GGGCATCAAGCAGCTCCAGGCAAGAATCCTGGCTGTGGAAAGATACCTAAAGGATCAACAGCT
CCTGGGGATTTGGGGTTGCTCTGGAAAACTCATTTGCACCACTGCTGTGCCTTGGAATGCTAG	CCTGGGGATTTGGGGTTGCTCTGGAAAACTCATTTGCACCACTGCTGTGCCTTGGAATGCTAG
TTGGAGTAATAAATCTCTGGAACAGATTTGGAATCACACGACCTGGATGGAGTGGGACAGAGA	TTGGAGTAATAAATCTCTGGAACAGATTTGGAATCACACGACCTGGATGGAGTGGGACAGAGA
AATTAACAATTACACAAGCTTAATACACTCCTTAATTGAAGAATCGCAAAACCAGCAAGAAAA	AATTAACAATTACACAAGCTTAATACACTCCTTAATTGAAGAATCGCAAAACCAGCAAGAAAA
GAATGAACAAGAATTATTGGAATTAGATAAATGGGCAAGTTTGTGGAATTGGTTTAACATAAC	GAATGAACAAGAATTATTGGAATTAGATAAATGGGCAAGTTTGTGGAATTGGTTTAACATAAC
AAATTGGCTGTGGTATATAAAATTATTCATAATGATAGTAGGAGGCTTGGTAGGTTTAAGAAT	AAATTGGCTGTGGTATATAAAATTATTCATAATGATAGTAGGAGGCTTGGTAGGTTTAAGAAT
AGTTTTTGCTGTACTTTCTATAGTGAATAGAGTTAGGCAGGGATATTCACCATTATCGTTTCA	AGTTTTTGCTGTACTTTCTATAGTGAATAGAGTTAGGCAGGGATATTCACCATTATCGTTTCA
GACCCACCTCCCAACCCCGAGGGGACCCGACAGGCCCGAAGGAATAGAAGAAGAAGGTGGAGA	GACCCACCTCCCAACCCCGAGGGGACCCGACAGGCCCGAAGGAATAGAAGAAGAAGGTGGAGA
GAGAGACAGAGACAGATCCATTCGATTAGTGAACGGATCTCGACGGTATCGATTAGACTGTAG	GAGAGACAGAGACAGATCCATTCGATTAGTGAACGGATCTCGACGGTATCGATTAGACTGTAG
CCCAGGAATATGGCAGCTAGATTGTACACATTTAGAAGGAAAAGTTATCTTGGTAGCAGTTCA	CCCAGGAATATGGCAGCTAGATTGTACACATTTAGAAGGAAAAGTTATCTTGGTAGCAGTTCA
TGTAGCCAGTGGATATATAGAAGCAGAAGTAATTCCAGCAGAGACAGGGCAAGAAACAGCATA	TGTAGCCAGTGGATATATAGAAGCAGAAGTAATTCCAGCAGAGACAGGGCAAGAAACAGCATA
CTTCCTCTTAAAATTAGCAGGAAGATGGCCAGTAAAAACAGTACATACAGACAATGGCAGCAA	CTTCCTCTTAAAATTAGCAGGAAGATGGCCAGTAAAAACAGTACATACAGACAATGGCAGCAA
TTTCACCAGTACTACAGTTAAGGCCGCCTGTTGGTGGGCGGGGATCAAGCAGGAATTTGGCAT	TTTCACCAGTACTACAGTTAAGGCCGCCTGTTGGTGGGCGGGGATCAAGCAGGAATTTGGCAT
TCCCTACAATCCCCAAAGTCAAGGAGTAATAGAATCTATGAATAAAGAATTAAAGAAAATTAT	TCCCTACAATCCCCAAAGTCAAGGAGTAATAGAATCTATGAATAAAGAATTAAAGAAAATTAT
AGGACAGGTAAGAGATCAGGCTGAACATCTTAAGACAGCAGTACAAATGGCAGTATTCATCCA	AGGACAGGTAAGAGATCAGGCTGAACATCTTAAGACAGCAGTACAAATGGCAGTATTCATCCA
CAATTTTAAAAGAAAAGGGGGGATTGGGGGGTACAGTGCAGGGGAAAGAATAGTAGACATAAT	CAATTTTAAAAGAAAAGGGGGGATTGGGGGGTACAGTGCAGGGGAAAGAATAGTAGACATAAT
AGCAACAGACATACAAACTAAAGAATTACAAAAACAAATTACAAAAATTCAAAATTTTCGGGT	AGCAACAGACATACAAACTAAAGAATTACAAAAACAAATTACAAAAATTCAAAATTTTCGGGT
TTATTACAGGGACAGCAGAGATCCAGTTTGGCTGCATACGCGTCGTGAGGCTCCGGTGCCCGT	TTATTACAGGGACAGCAGAGATCCAGTTTGGCTGCATACGCGTCGTGAGGCTCCGGTGCCCGT
CAGTGGGCAGAGCGCACATCGCCCACAGTCCCCGAGAAGTTGGGGGGAGGGGTCGGCAATTGA	CAGTGGGCAGAGCGCACATCGCCCACAGTCCCCGAGAAGTTGGGGGGAGGGGTCGGCAATTGA
ACCGGTGCCTAGAGAAGGTGGCGCGGGGTAAACTGGGAAAGTGATGTCGTGTACTGGCTCCGC	ACCGGTGCCTAGAGAAGGTGGCGCGGGGTAAACTGGGAAAGTGATGTCGTGTACTGGCTCCGC
CTTTTTCCCGAGGGTGGGGGAGAACCGTATATAAGTGCAGTAGTCGCCGTGAACGTTCTTTTT	CTTTTTCCCGAGGGTGGGGGAGAACCGTATATAAGTGCAGTAGTCGCCGTGAACGTTCTTTTT
CGCAACGGGTTTGCCGCCAGAACACAGGTAAGTGCCGTGTGTGGTTCCCGCGGGCCTGGCCTC	CGCAACGGGTTTGCCGCCAGAACACAGGTAAGTGCCGTGTGTGGTTCCCGCGGGCCTGGCCTC
TTTACGGGTTATGGCCCTTGCGTGCCTTGAATTACTTCCACCTGGCTGCAGTACGTGATTCTT	TTTACGGGTTATGGCCCTTGCGTGCCTTGAATTACTTCCACCTGGCTGCAGTACGTGATTCTT
GATCCCGAGCTTCGGGTTGGAAGTGGGTGGGAGAGTTCGAGGCCTTGCGCTTAAGGAGCCCCT	GATCCCGAGCTTCGGGTTGGAAGTGGGTGGGAGAGTTCGAGGCCTTGCGCTTAAGGAGCCCCT
TCGCCTCGTGCTTGAGTTGAGGCCTGGCCTGGGCGCTGGGGCCGCCGCGTGCGAATCTGGTGG	TCGCCTCGTGCTTGAGTTGAGGCCTGGCCTGGGCGCTGGGGCCGCCGCGTGCGAATCTGGTGG
CACCTTCGCGCCTGTCTCGCTGCTTTCGATAAGTCTCTAGCCATTTAAAATTTTTGATGACCT	CACCTTCGCGCCTGTCTCGCTGCTTTCGATAAGTCTCTAGCCATTTAAAATTTTTGATGACCT
GCTGCGACGCTTTTTTTCTGGCAAGATAGTCTTGTAAATGCGGGCCAAGATCTGCACACTGGT	GCTGCGACGCTTTTTTTCTGGCAAGATAGTCTTGTAAATGCGGGCCAAGATCTGCACACTGGT
ATTTCGGTTTTTGGGGCCGCGGGCGGCGACGGGGCCCGTGCGTCCCAGCGCACATGTTCGGCG	ATTTCGGTTTTTGGGGCCGCGGGCGGCGACGGGGCCCGTGCGTCCCAGCGCACATGTTCGGCG
AGGCGGGGCCTGCGAGCGCGGCCACCGAGAATCGGACGGGGGTAGTCTCAAGCTGGCCGGCCT	AGGCGGGGCCTGCGAGCGCGGCCACCGAGAATCGGACGGGGGTAGTCTCAAGCTGGCCGGCCT
GCTCTGGTGCCTGGCCTCGCGCCGCCGTGTATCGCCCCGCCCTGGGCGGCAAGGCTGGCCCGG	GCTCTGGTGCCTGGCCTCGCGCCGCCGTGTATCGCCCCGCCCTGGGCGGCAAGGCTGGCCCGG
TCGGCACCAGTTGCGTGAGCGGAAAGATGGCCGCTTCCCGGCCCTGCTGCAGGGAGCTCAAAA	TCGGCACCAGTTGCGTGAGCGGAAAGATGGCCGCTTCCCGGCCCTGCTGCAGGGAGCTCAAAA
TGGAGGACGCGGCGCTCGGGAGAGCGGGCGGGTGAGTCACCCACACAAAGGAAAAGGGCCTTT	TGGAGGACGCGGCGCTCGGGAGAGCGGGCGGGTGAGTCACCCACACAAAGGAAAAGGGCCTTT
CCGTCCTCAGCCGTCGCTTCATGTGACTCCACTGAGTACCGGGCGCCGTCCAGGCACCTCGAT	CCGTCCTCAGCCGTCGCTTCATGTGACTCCACTGAGTACCGGGCGCCGTCCAGGCACCTCGAT
TAGTTCTCGTGCTTTTGGAGTACGTCGTCTTTAGGTTGGGGGGAGGGGTTTTATGCGATGGAG	TAGTTCTCGTGCTTTTGGAGTACGTCGTCTTTAGGTTGGGGGGAGGGGTTTTATGCGATGGAG
TTTCCCCACACTGAGTGGGTGGAGACTGAAGTTAGGCCAGCTTGGCACTTGATGTAATTCTCC	TTTCCCCACACTGAGTGGGTGGAGACTGAAGTTAGGCCAGCTTGGCACTTGATGTAATTCTCC
TTGGAATTTGCCCTTTTTGAGTTTGGATCTTGGTTCATTCTCAAGCCTCAGACAGTGGTTCAA	TTGGAATTTGCCCTTTTTGAGTTTGGATCTTGGTTCATTCTCAAGCCTCAGACAGTGGTTCAA
AGTTTTTTTCTTCCATTTCAGGTGTCGTGAGCTAGCTCTAGAGCCACCATGGCCTTACCAGTG	AGTTTTTTTCTTCCATTTCAGGTGTCGTGAGCTAGCTCTAGAGCCACCATGGCCTTACCAGTG
ACCGCCTTGCTCCTGCCGCTGGCCTTGCTGCTCCACGCCGCCAGGCCGGAGGCTGTTATGACA	ACCGCCTTGCTCCTGCCGCTGGCCTTGCTGCTCCACGCCGCCAGGCCGGAGGCTGTTATGACA
CAGACCCCTCTCTCCCTGGCTGTGACACCTGGCGAGCTGGCCACCATCAGCTGTAGAGCCAGC	CAGACCCCTCTCTCCCTGGCTGTGACACCTGGCGAGCTGGCCACCATCAGCTGTAGAGCCAGC
CAGAGCCTGCTGCACAGAGACGGAAAAAGCTACCTGAGCTGGTATCTGCAAAAGCCCGGACAG	CAGAGCCTGCTGCACAGAGACGGAAAAAGCTACCTGAGCTGGTATCTGCAAAAGCCCGGACAG
GCCCCTAGACCTCTGATCTACGAGGCCAGCAAGCGGTTTAGCGGCGTGTCTGATAGATTCAGC	GCCCCTAGACCTCTGATCTACGAGGCCAGCAAGCGGTTTAGCGGCGTGTCTGATAGATTCAGC
GGCTCCGGCTCTGGCACCGACTTCACACTGAAGATCAGCAGAGTGGAAGCTGAAGATGTGGGC	GGCTCCGGCTCTGGCACCGACTTCACACTGAAGATCAGCAGAGTGGAAGCTGAAGACGTGGGC
GTGTACTATTGCCAGCAGTCTCTGCACTTCCCACCTACATTCGGCCAGGGCACCAAGGTGGAA	GTGTACTATTGCCAGCAGTCTCTGCACTTCCCACCTACATTCGGCCAGGGCACCAAGGTGGAA
ATCAAGGGCGGCAGCAGCAGAAGCAGCAGCTCTGGCGGAGGCGGCAGCGGAGGCGGCGGTGAG	ATCAAGGGCGGCAGCAGCAGAAGCAGCAGCTCTGGCGGAGGCGGCAGCGGAGGCGGCGGTGAG
GTGCCCCTGGTGGAGAGCGGAGGAGATCTGGTCAAGCCTGGCGGATCTCTGCGGCTGAGCTGC	GTGCCCCTGGTGGAGAGCGGAGGAGATCTGGTCAAGCCTGGCGGATCTCTGCGGCTGAGCTGC
ATCAGCAGTGGCTTCACCTTTAGCGACTACGGCATGACCTGGGTGCGGCAGAGCCCCGGCAAA	ATCAGCAGTGGCTTCACCTTTAGCGACTACGGCATGACCTGGGTGCGGCAGAGCCCCGGCAAA
GGCCTGCAGTGGGTCGCCTCTATCCGGCGGGACGGCAGCGACACATACTACACCGACGCCGTG	GGCCTGCAGTGGGTCGCCTCTATCCGGCGGGACGGCAGCGACACATACTACACCGACGCCGTG
GAAGGCAGATTCACCATTTCTAGGGACAACGCCAACAACACCCTGTACCTTCAAATGAACAGC	GAAGGCAGATTCACCATTTCTAGGGACAACGCCAACAACACCCTGTACCTTCAAATGAACAGC
CTGAGAGCCGAGGACACCGCCGTGTACTACTGCGCCAGAGATGGCAACAATTGGTCCTGGGAC	CTGAGAGCCGAGGACACCGCCGTGTACTACTGCGCCAGAGACGGCAACAATTGGTCCTGGGAC
TACTGGGGCCACGGCACCAGCCTGTTCGTGTCCTCCACCACGACGCCAGCGCCGCGACCACCA	TACTGGGGCCACGGCACCAGCCTGTTCGTGTCCTCCACCACGACGCCAGCGCCGCGACCACCA
ACACCGGCGCCCACCATCGCGTCGCAGCCCCTGTCCCTGCGCCCAGAGGCGTGCCGGCCAGCG	ACACCGGCGCCCACCATCGCGTCGCAGCCCCTGTCCCTGCGCCCAGAGGCGTGCCGGCCAGCG
GCGGGGGGCGCAGTGCACACGAGGGGGCTGGACTTCGCCTGTGATATCTACATCTGGGCGCCC	GCGGGGGGCGCAGTGCACACGAGGGGGCTGGACTTCGCCTGTGATATCTACATCTGGGCGCCC
TTGGCCGGGACTTGTGGGGTCCTTCTCCTGTCACTGGTTATCACCCTTTACTGCAAACGGGGC	TTGGCCGGGACTTGTGGGGTCCTTCTCCTGTCACTGGTTATCACCCTTTACTGCAAACGGGGC
AGAAAGAAACTCCTGTATATATTCAAACAACCATTTATGAGACCAGTACAAACTACTCAAGAG	AGAAAGAAACTCCTGTATATATTCAAACAACCATTTATGAGACCAGTACAAACTACTCAAGAG
GAAGATGGCTGTAGCTGCCGATTTCCAGAAGAAGAAGAAGGAGGATGTGAACTGAGAGTGAAG	GAAGATGGCTGTAGCTGCCGATTTCCAGAAGAAGAAGAAGGAGGATGTGAACTGAGAGTGAAG
TTCAGCAGGAGCGCAGACGCCCCCGCGTACAAGCAGGGCCAGAACCAGCTCTATAACGAGCTC	TTCAGCAGGAGCGCAGACGCCCCCGCGTACAAGCAGGGCCAGAACCAGCTCTATAACGAGCTC
AATCTAGGACGAAGAGAGGAGTACGATGTTTTGGACAAGAGACGTGGCCGGGACCCTGAGATG	AATCTAGGACGAAGAGAGGAGTACGATGTTTTGGACAAGAGACGTGGCCGGGACCCTGAGATG
GGGGGAAAGCCGAGAAGGAAGAACCCTCAGGAAGGCCTGTACAATGAACTGCAGAAAGATAAG	GGGGGAAAGCCGAGAAGGAAGAACCCTCAGGAAGGCCTGTACAATGAACTGCAGAAAGATAAG
ATGGCGGAGGCCTACAGTGAGATTGGGATGAAAGGCGAGCGCCGGAGGGGCAAGGGGCACGAT	ATGGCGGAGGCCTACAGTGAGATTGGGATGAAAGGCGAGCGCCGGAGGGGCAAGGGGCACGAC
GGCCTTTACCAGGGTCTCAGTACAGCCACCAAGGACACCTACGACGCCCTTCACATGCAGGCC	GGCCTTTACCAGGGTCTCAGTACAGCCACCAAGGACACCTACGACGCCCTTCACATGCAGGCC
CTGCCCCCTCGCTAACGTCGACAATCAACCTCTGGATTACAAAATTTGTGAAAGATTGACTGG	CTGCCCCCTCGCTAAGTCGACAATCAACCTCTGGATTACAAAATTTGTGAAAGATTGACTGGT
TATTCTTAACTATGTTGCTCCTTTTACGCTATGTGGATACGCTGCTTTAATGCCTTTGTATCA	ATTCTTAACTATGTTGCTCCTTTTACGCTATGTGGATACGCTGCTTTAATGCCTTTGTATCAT
TGCTATTGCTTCCCGTATGGCTTTCATTTTCTCCTCCTTGTATAAATCCTGGTTGCTGTCTCT	GCTATTGCTTCCCGTATGGCTTTCATTTTCTCCTCCTTGTATAAATCCTGGTTGCTGTCTCTT
TTATGAGGAGTTGTGGCCCGTTGTCAGGCAACGTGGCGTGGTGTGCACTGTGTTTGCTGACGC	TATGAGGAGTTGTGGCCCGTTGTCAGGCAACGTGGCGTGGTGTGCACTGTGTTTGCTGACGCA
AACCCCCACTGGTTGGGGCATTGCCACCACCTGTCAGCTCCTTTCCGGGACTTTCGCTTTCCC	ACCCCCACTGGTTGGGGCATTGCCACCACCTGTCAGCTCCTTTCCGGGACTTTCGCTTTCCCC
CCTCCCTATTGCCACGGCGGAACTCATCGCCGCCTGCCTTGCCCGCTGCTGGACAGGGGCTCG	CTCCCTATTGCCACGGCGGAACTCATCGCCGCCTGCCTTGCCCGCTGCTGGACAGGGGCTCGG
GCTGTTGGGCACTGACAATTCCGTGGTGTTGTCGGGGAAGCTGACGTCCTTTCCTTGGCTGCT	CTGTTGGGCACTGACAATTCCGTGGTGTTGTCGGGGAAGCTGACGTCCTTTCCTTGGCTGCTC
CGCCTGTGTTGCCACCTGGATTCTGCGCGGGACGTCCTTCTGCTACGTCCCTTCGGCCCTCAA	GCCTGTGTTGCCACCTGGATTCTGCGCGGGACGTCCTTCTGCTACGTCCCTTCGGCCCTCAAT
TCCAGCGGACCTTCCTTCCCGCGGCCTGCTGCCGGCTCTGCGGCCTCTTCCGCGTCTTCGCCT	CCAGCGGACCTTCCTTCCCGCGGCCTGCTGCCGGCTCTGCGGCCTCTTCCGCGTCTTCGCCTT
TCGCCCTCAGACGAGTCGGATCTCCCTTTGGGCCGCCTCCCCGCCTGGAATTCGAGCTCGGTA	CGCCCTCAGACGAGTCGGATCTCCCTTTGGGCCGCCTCCCCGCCTGGAATTCGAGCTCGGTAC
CCTTTAAGACCAATGACTTACAAGGCAGCTGTAGATCTTAGCCACTTTTTAAAAGAAAAGGGG	CTTTAAGACCAATGACTTACAAGGCAGCTGTAGATCTTAGCCACTTTTTAAAAGAAAAGGGGG
GGACTGGAAGGGCTAATTCACTCCCAACGAAGACAAGATCTGCTTTTTGCTTGTACTGGGTCT	GACTGGAAGGGCTAATTCACTCCCAACGAAGACAAGATCTGCTTTTTGCTTGTACTGGGTCTC
CTCTGGTTAGACCAGATCTGAGCCTGGGAGCTCTCTGGCTAACTAGGGAACCCACTGCTTAAG	TCTGGTTAGACCAGATCTGAGCCTGGGAGCTCTCTGGCTAACTAGGGAACCCACTGCTTAAGC
CCTCAATAAAGCTTGCCTTGAGTGCTTCAAGTAGTGTGTGCCCGTCTGTTGTGTGACTCTGGT	CTCAATAAAGCTTGCCTTGAGTGCTTCAAGTAGTGTGTGCCCGTCTGTTGTGTGACTCTGGTA
AACTAGAGATCCCTCAGACCCTTTTAGTCAGTGTGGAAAATCTCTAGCAGTAGTAGTTCATGT	ACTAGAGATCCCTCAGACCCTTTTAGTCAGTGTGGAAAATCTCTAGCAGTAGTAGTTCATGTC
CATCTTATTATTCAGTATTTATAACTTGCAAAGAAATGAATATCAGAGAGTGAGAGGAACTTG	ATCTTATTATTCAGTATTTATAACTTGCAAAGAAATGAATATCAGAGAGTGAGAGGAACTTGT
TTTATTGCAGCTTATAATGGTTACAAATAAAGCAATAGCATCACAAATTTCACAAATAAAGCA	TTATTGCAGCTTATAATGGTTACAAATAAAGCAATAGCATCACAAATTTCACAAATAAAGCAT
TTTTTTTCACTGCATTCTAGTTGTGGTTTGTCCAAACTCATCAATGTATCTTATCATGTCTGG	TTTTTTCACTGCATTCTAGTTGTGGTTTGTCCAAACTCATCAATGTATCTTATCATGTCTGGC
CTCTAGCTATCCCGCCCCTAACTCCGCCCAGTTCCGCCCATTCTCCGCCCCATGGCTGACTAA	TCTAGCTATCCCGCCCCTAACTCCGCCCAGTTCCGCCCATTCTCCGCCCCATGGCTGACTAAT
TTTTTTTTATTTATGCAGAGGCCGAGGCCGCCTCGGCCTCTGAGCTATTCCAGAAGTAGTGAG	TTTTTTTATTTATGCAGAGGCCGAGGCCGCCTCGGCCTCTGAGCTATTCCAGAAGTAGTGAGG
GAGGCTTTTTTGGAGGCCTAGCTAGGGACGTACCCAATTCGCCCTATAGTGAGTCGTATTACG	AGGCTTTTTTGGAGGCCTAGCTAGGGACGTACCCAATTCGCCCTATAGTGAGTCGTATTACGC
CGCGCTCACTGGCCGTCGTTTTACAACGTCGTGACTGGGAAAACCCTGGCGTTACCCAACTTA	GCGCTCACTGGCCGTCGTTTTACAACGTCGTGACTGGGAAAACCCTGGCGTTACCCAACTTAA
ATCGCCTTGCAGCACATCCCCCTTTCGCCAGCTGGCGTAATAGCGAAGAGGCCCGCACCGATC	TCGCCTTGCAGCACATCCCCCTTTCGCCAGCTGGCGTAATAGCGAAGAGGCCCGCACCGATCG
GCCCTTCCCAACAGTTGCGCAGCCTGAATGGCGAATGGACGCGCCCTGTAGCGGCGCATTAA	CCCTTCCCAACAGTTGCGCAGCCTGAATGGCGAATGGACGCGCCCTGTAGCGGCGCATTAAG
GCGCGGCGGGTGTGGTGGTTACGCGCAGCGTGACCGCTACACTTGCCAGCGCCCTAGCGCCCG	CGCGGCGGGTGTGGTGGTTACGCGCAGCGTGACCGCTACACTTGCCAGCGCCCTAGCGCCCGC
CTCCTTTCGCTTTCTTCCCTTCCTTTCTCGCCACGTTCGCCGGCTTTCCCCGTCAAGCTCTAA	TCCTTTCGCTTTCTTCCCTTCCTTTCTCGCCACGTTCGCCGGCTTTCCCCGTCAAGCTCTAAA
ATCGGGGGCTCCCTTTAGGGTTCCGATTTAGTGCTTTACGGCACCTCGACCCCAAAAAACTTG	TCGGGGGCTCCCTTTAGGGTTCCGATTTAGTGCTTTACGGCACCTCGACCCCAAAAAACTTGA
ATTAGGGTGATGGTTCACGTAGTGGGCCATCGCCCTGATAGACGGTTTTTCGCCCTTTGACGT	TTAGGGTGATGGTTCACGTAGTGGGCCATCGCCCTGATAGACGGTTTTTCGCCCTTTGACGTT
TGGAGTCCACGTTCTTTAATAGTGGACTCTTGTTCCAAACTGGAACAACACTCAACCCTATCT	GGAGTCCACGTTCTTTAATAGTGGACTCTTGTTCCAAACTGGAACAACACTCAACCCTATCTC
CGGTCTATTCTTTTGATTTATAAGGGATTTTGCCGATTTCGGCCTATTGGTTAAAAAATGAGC	GGTCTATTCTTTTGATTTATAAGGGATTTTGCCGATTTCGGCCTATTGGTTAAAAAATGAGCT
TGATTTAACAAAAATTTAACGCGAATTTTAACAAAATATTAACGCTTACAATTTAGGTGGCAC	GATTTAACAAAAATTTAACGCGAATTTTAACAAAATATTAACGCTTACAATTTAGGTGGCACT
TTTTCGGGGAAATGTGCGCGGAACCCCTATTTGTTTATTTTTCTAAATACATTCAAATATGTA	TTTCGGGGAAATGTGCGCGGAACCCCTATTTGTTTATTTTTCTAAATACATTCAAATATGTAT
TCCGCTCATGAGACAATAACCCTGATAAATGCTTCAATAATATTGAAAAAGGAAGAGTATGAG	CCGCTCATGAGACAATAACCCTGATAAATGCTTCAATAATATTGAAAAAGGAAGAGTATGAGT
TATTCAACATTTCCGTGTCGCCCTTATTCCCTTTTTTGCGGCATTTTGCCTTCCTGTTTTTGC	ATTCAACATTTCCGTGTCGCCCTTATTCCCTTTTTTGCGGCATTTTGCCTTCCTGTTTTTGCT
TCACCCAGAAACGCTGGTGAAAGTAAAAGATGCTGAAGATCAGTTGG	CACCCAGAAACGCTGGTGAAAGTAAAAGATGCTGAAGATCAGTTGG

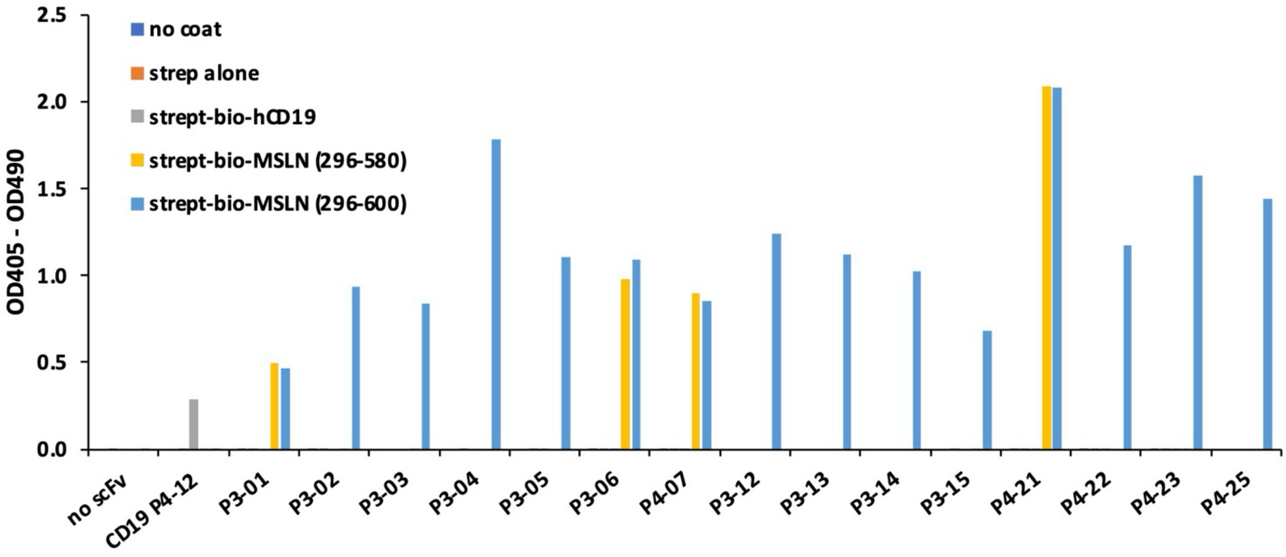

Supplementary Figure 1. MSLN binding patterns of scFvs derived from phage library panning campaign. Representative phage ELISA showing binding of 15 (of 66) randomly selected scFv clones to MSLN (296-600) and MSLN (296-580) polypeptides representing full-length and shed domains, respectively. Results for this representative group show that P3-01, P3-06, P4-07, and P4-21 bind both MSLN polypeptides suggesting specificity for shed MSLN while the rest bind only MSLN (296-600) which contains the stump domain. Controls included uncoated microplate wells and wells coated with only streptavidin (strep) or streptavidin and biotinylated human CD19 protein, as well as a phage displayed anti-human CD19 scFv isolated from the canine phage library in a different panning campaign.

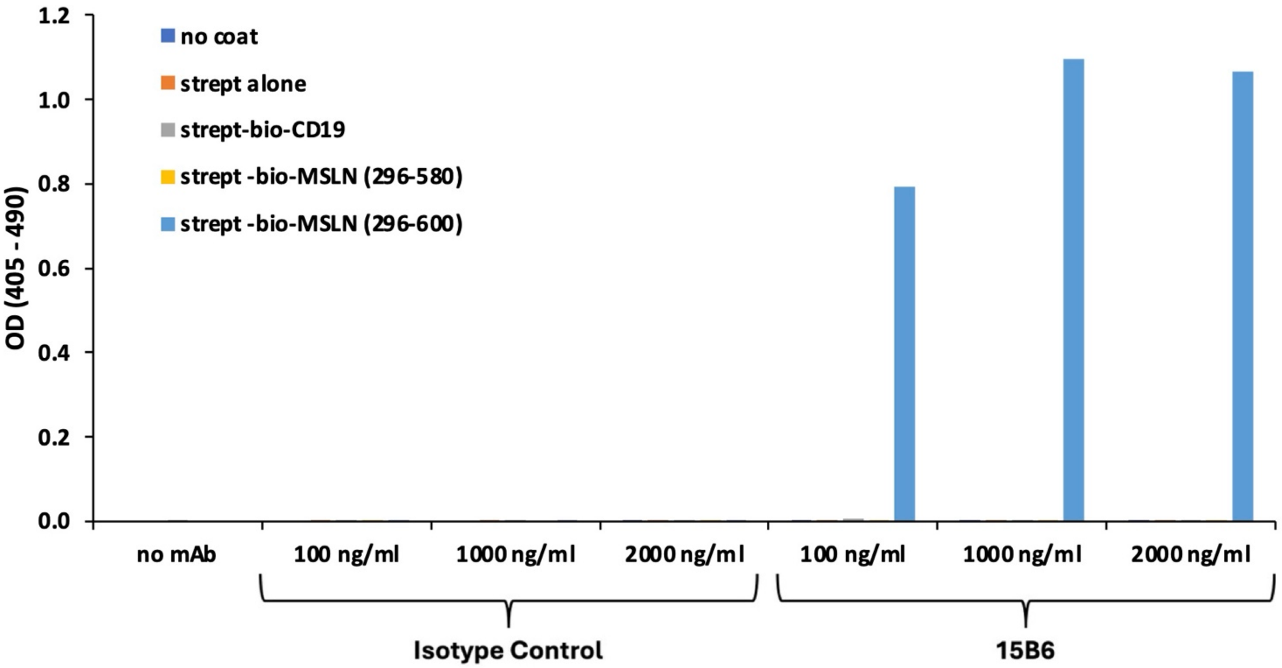

Supplementary Figure 2. Binding of recombinant murine 15B6 to MSLN polypeptides. ELISA to verify that recombinantly-produced 15B6 reference antibody retains binding to MSLN stump region. Microplate wells were uncoated or coated with streptavidin (strept) alone, or streptavidin and biotinylated human CD19, MSLN (296-580), and MSLN (296-600) and reacted with recombinant 15B6 or a murine isotype control (anti-dinitrophenol). Recombinant 15B6 binds only stump-containing full-length MSLN (296-600) but not truncated MSLN (296-580).

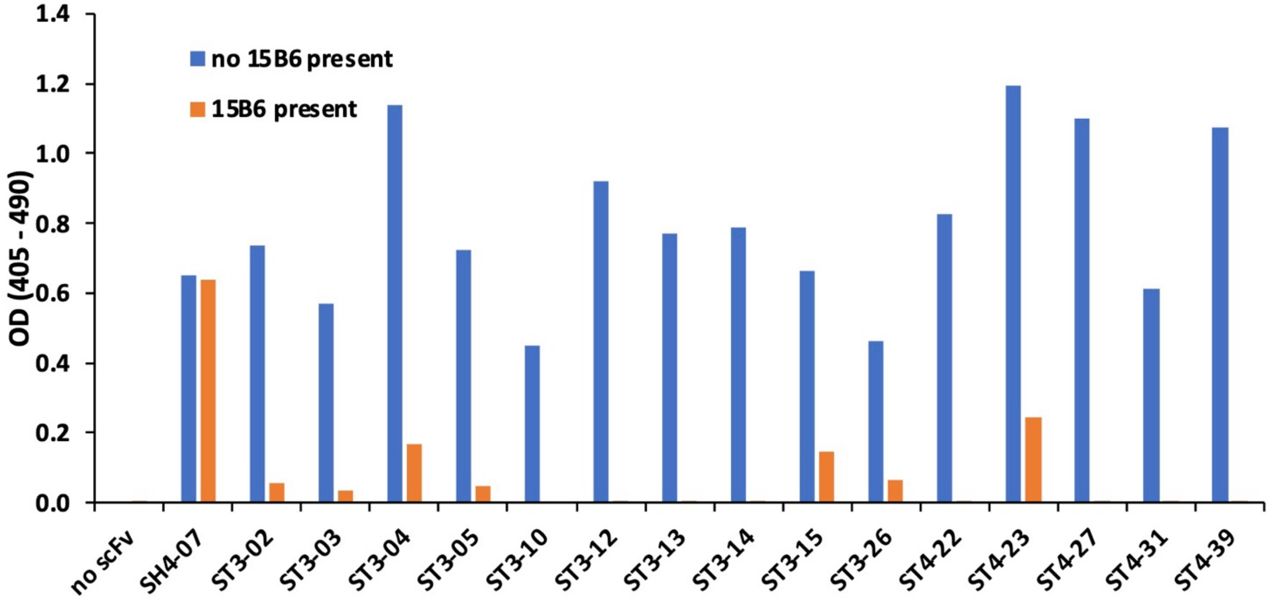

Supplementary Figure 3. 15B6 competition ELISA. Representative MSLN (296-600) binding profiles shown for 15 of 29 stump-binding phage display-scFvs in presence or absence of pre-bound 15B6 reference antibody. Ratios of binding in the absence to the binding in the presence of 15B6 were calculated from these data for all 29 scFvs and listed in **Figure 2c**. Internal control scFv SH4-07, shown to be directed to shed MSLN polypeptide **(Supplementary Figure 1)**, shows no inhibition by stump-directed 15B6 as expected.

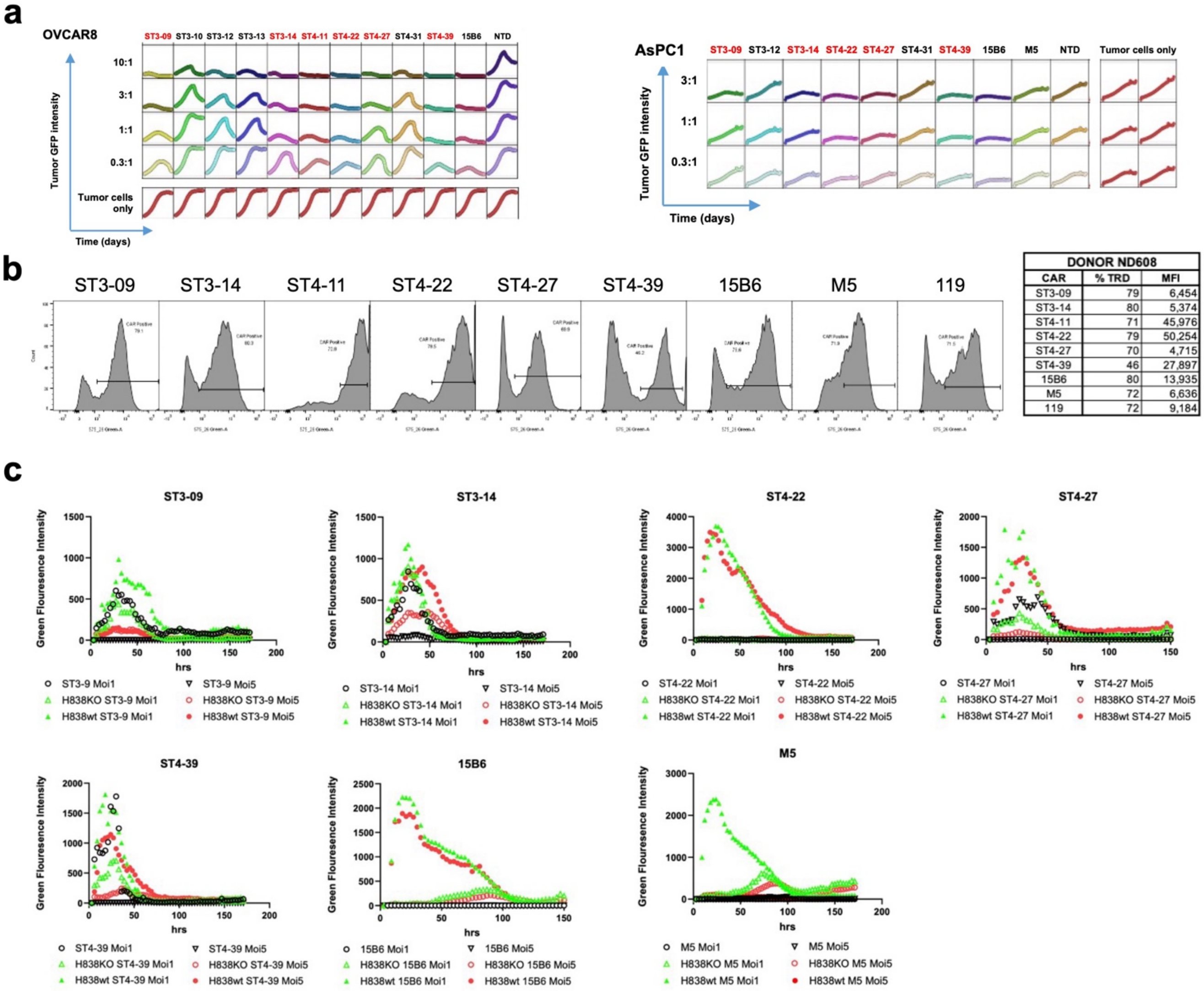

DONOR ND608		
CAR	% TRD	MFI
ST3-09	79	6,454
ST3-14	80	5,374
ST4-11	71	45,976
ST4-22	79	50,254
ST4-27	70	4,715
ST4-39	46	27,897
15B6	80	13,935
M5	72	6,636
119	72	9,184

Supplementary Figure 4. Expression and activation properties of MSLN stump-directed CAR-T cells. (a) Initial group of 10 ST-scFvs were screened in human CAR T cells in a 96-well Incucyte® cell killing assay targeting MSLN expressed on either OVCAR8 or AsPC1 cells. Each candidate was screened at an E:T ratio of 10:1, 3:1, 1:1 or 0.3:1 with tumor GFP intensities measured every 3 h over a 7-day period. (b) Flow histograms of healthy donor ND608 T cells showing expression of MSLN-directed and CD19-directed (119, control) CARs. At Day 8 near the end of CAR-T expansion, cell-surface scFvs were detected as follows: ST series with biotinylated rabbit anti-canine IgG, 15B6 was stained with biotinylated goat anti-murine IgG, and M5 and 119 (CD19 control) were stained with biotinylated goat anti-human IgG followed by phycoerythrin-labeled streptavidin. Percent transduction (% TRD) and surface mean fluorescence intensity (MFI) are tabulated. (c) Evaluation of CAR stump binder activation from Jurkat NFAT-GFP reporter cells expressing CARs. Reporter cells were transduced at a multiplicity of infection (Moi) of 1 or 5 lentiviral transduction units per Jurkat cell to determine the effect of cell surface CAR density during T cell activation in the presence and absence of antigen. ST3-09, ST3-14 and ST4-39 in the absence of antigen show greater tonic activity at

an Moi of 1. No apparent tonic signaling is seen with ST4-22, 15B6 or M5. Jurkat reporter CAR T cells were co-cultured with either H838 MSLN-KO or H838 wild type cells at an E:T ratio of 1:1. Plotting of data was normalized to those data at the 3-hour time point except for “15B6 Moi1” and M5 “H838wt Moi5” (closed red circles) where normalization was to 0-hour values due to an instrument failure for those wells at the 3-hour timepoint.

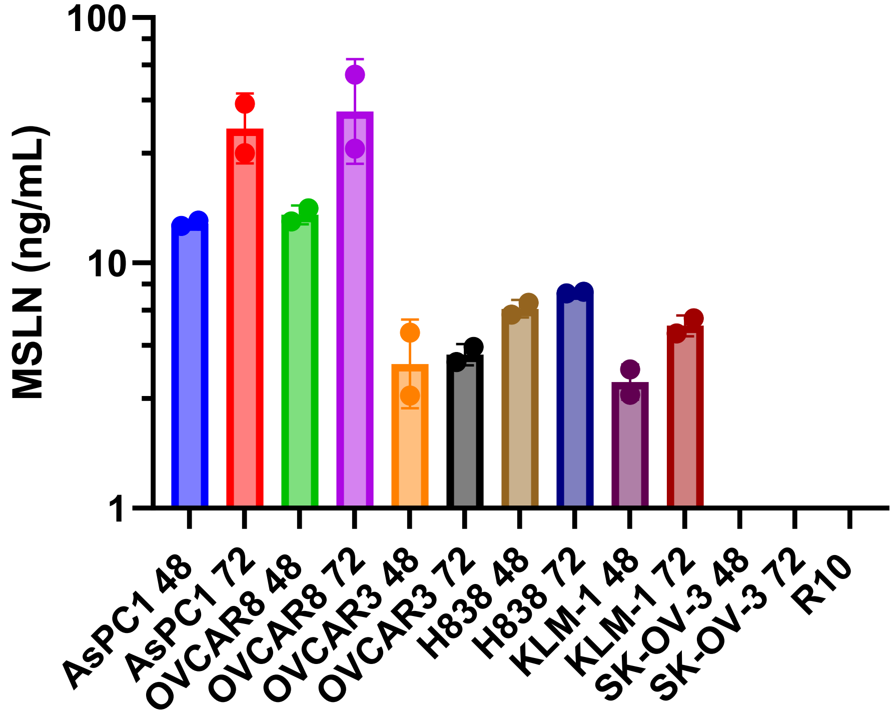

Supplementary Figure 5. Soluble MSLN production from cultured tumor cell lines. Tumor cell lines as indicated on horizontal axis were seeded at 1e6 cells per 2 ml/well of 6-well plates and confirmed to be 90% confluent at 24 hours. At the indicated time points (48 and 72 hours), 200 µL of culture supernatants were collected, centrifuged to remove cellular debris, diluted 4-fold in sample buffer, and evaluated by MSLN ELISA. R10 is RPMI 1640 media with 10% fetal calf serum.

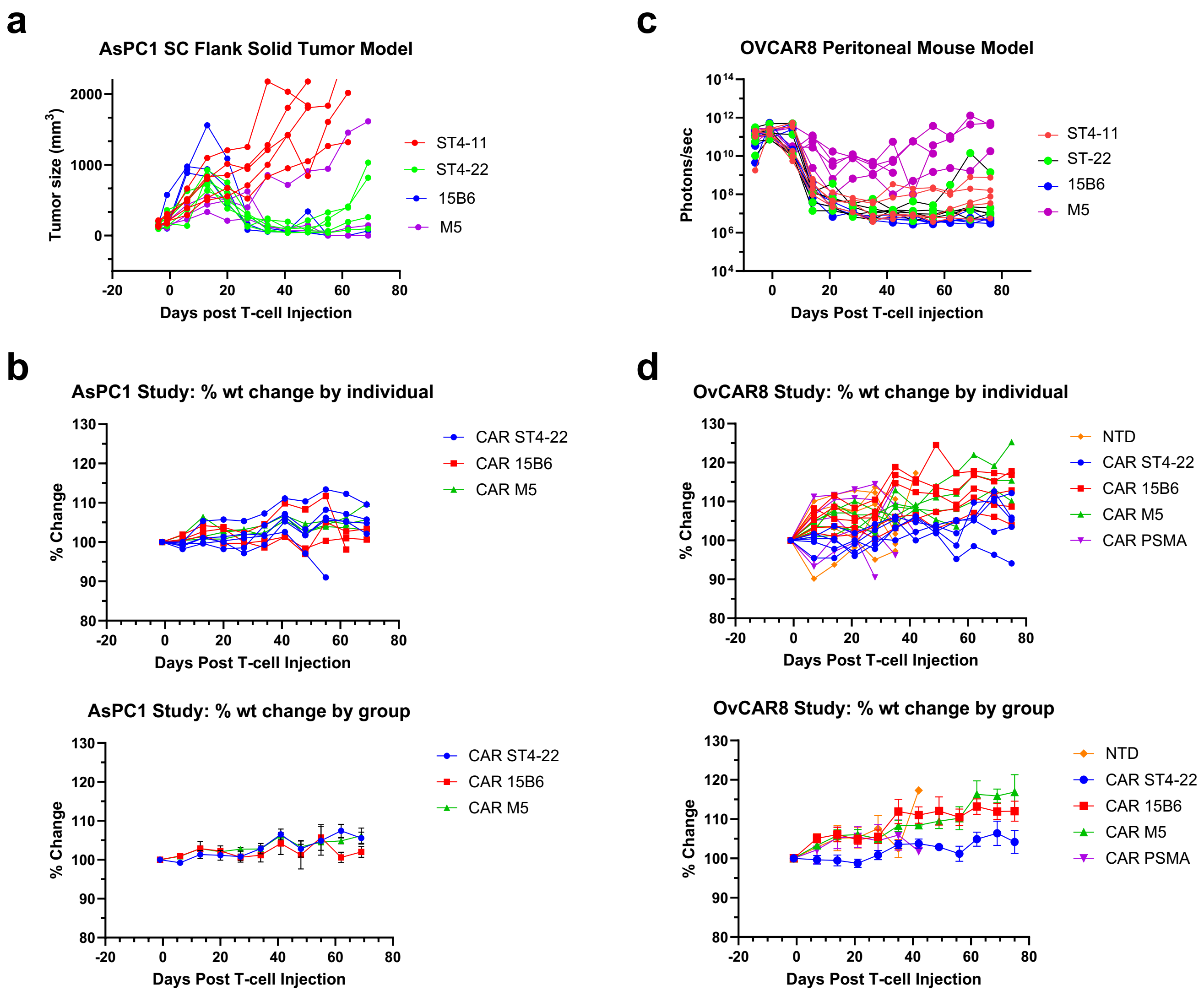

Supplementary Figure 6. Tumor control and weight of individual mice from in vivo study (Figure 3 d and e). (a) For AsPC1 tumor model, tumor size of individual mice treated with top stump CAR candidates illustrate scatter of tumor control. **(b)** corresponding body weight change over time of individual mouse (upper panel) and by group mean (lower panel). **(c)** For OVCAR8 tumor model, tumor BLIs of individual mice treated with top stump CAR candidates illustrate scatter of tumor control. (d) corresponding body weight change over time of individual mouse (upper panel) and by group mean (lower panel).

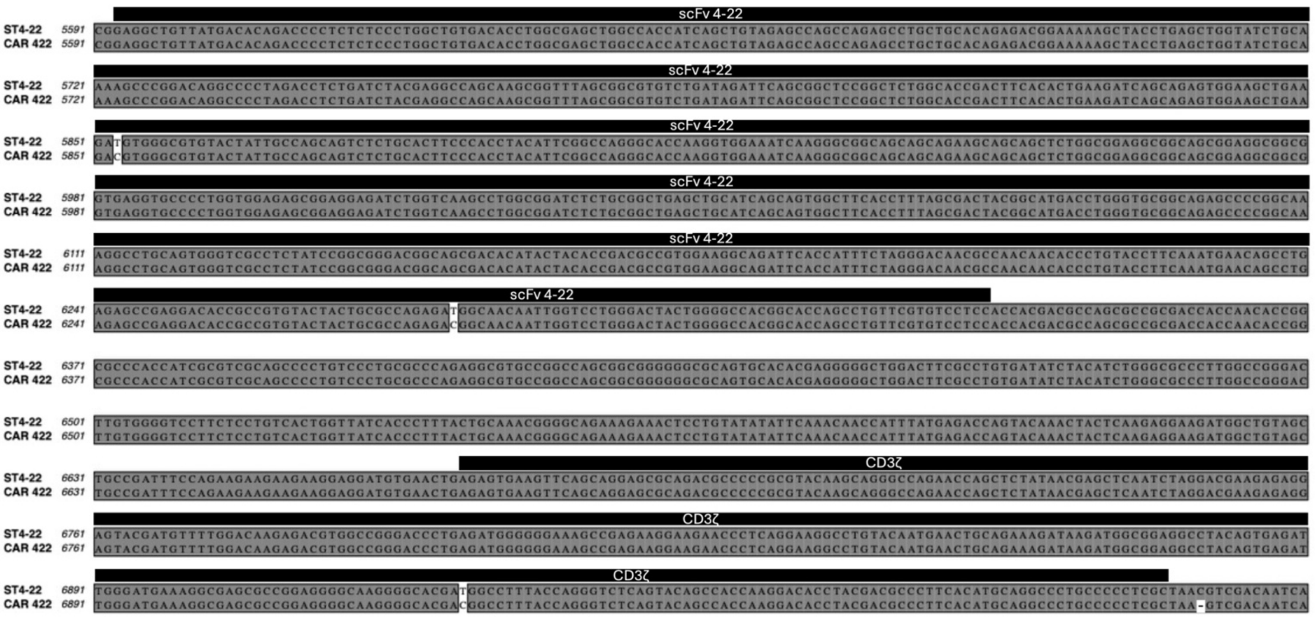

Supplementary Figure 7. Nucleotide alignment of CAR vectors for ST4-22 and CAR 422 to show elimination of internal ATG start sites from alternate reading frames and deletion of nucleotide downstream of CAR CDS. Locations for the 4 modifications within vector nucleotide positions 5591 and 7020 are shown along with positions encoding scFv 4-22 and CD3ζ domains.

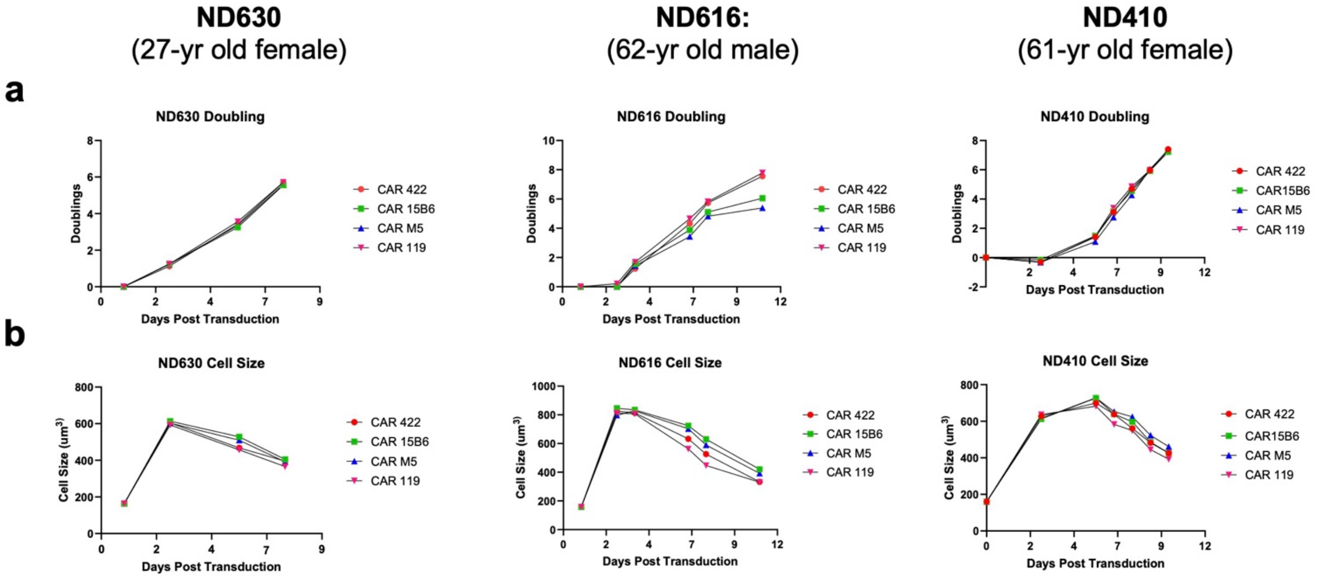

c

	DONOR ND630		DONOR ND616		DONOR ND410	
CAR	% TRD	MFI	% TRD	MFI	% TRD	MFI
422	63	32,908	70	18,442	61	19,576
15B6	58	16,641	74	20,768	37	49,203
M5	34	1,601	43	1,393	35	2,848
119	20	4,193	34	3,664	30	40,611

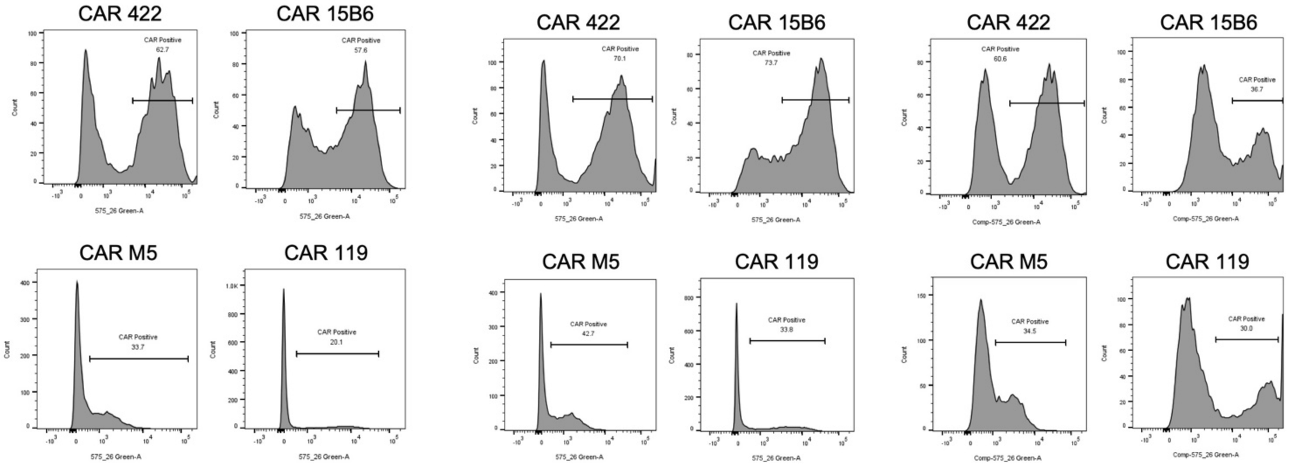

Supplementary Figure 8. CAR T cell manufacturing profiles in cells from three healthy human donors. MSLN-directed CAR T cells 422, 15B6, and M5, and CD19-directed CAR 119 were transduced into three healthy donors ND630, ND616 and ND410. **(a)** number of doublings and **(b)** change in cell size are compared. (c) Percent transduction efficiency (% TRD) and mean fluorescent intensity (MFI) are tabulated and displayed in histograms.

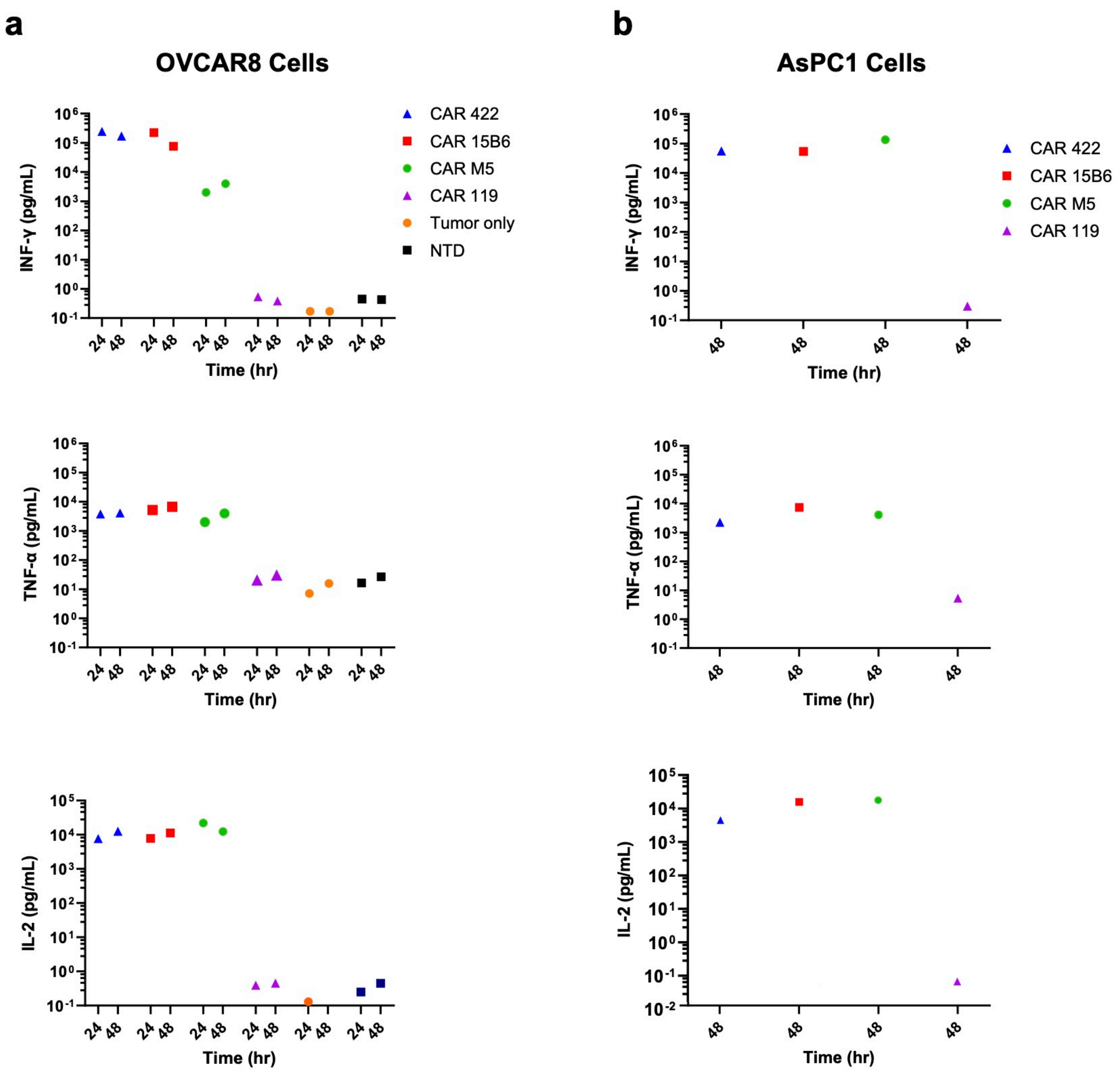

Supplementary Figure 9. CAR T cytokine production in co-cultures with OVCAR8 and AsPC1 cells. CAR+ T cells (1e6) manufactured from T cell donor ND410 (as used in Figure 4d) were co-cultured at a 1:1 E:T ratio with **(a)** OVCAR8 cells or **(b)** AsPC1 cells in 2 mL per well of a 6-well plate. At 24 and/or 48 hours (as indicated on horizontal axes), 200μL of media were removed for cytokine measurement. The profile of INF-γ, TNF-α, and IL-2 levels are plotted (top, middle, lower panels, respectively).

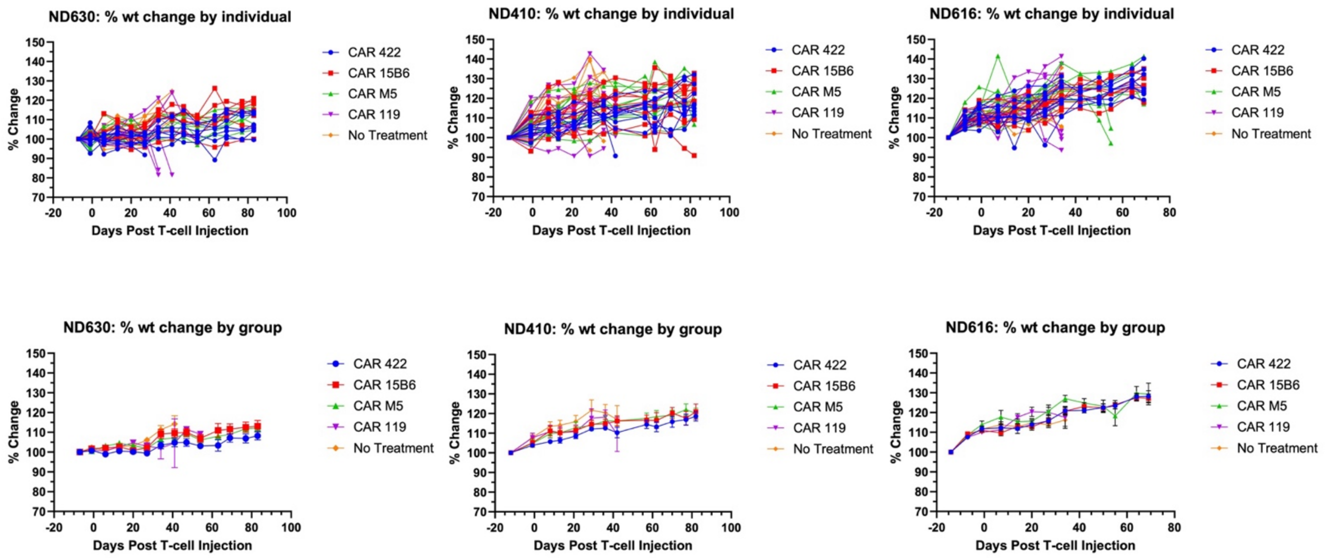

Supplementary Figure 10. Body weight change over time. Shown are data for individual mice (upper panels) and group means (lower panels) for OVCAR8 NSG mouse studies detailed in **Figure 4d**.

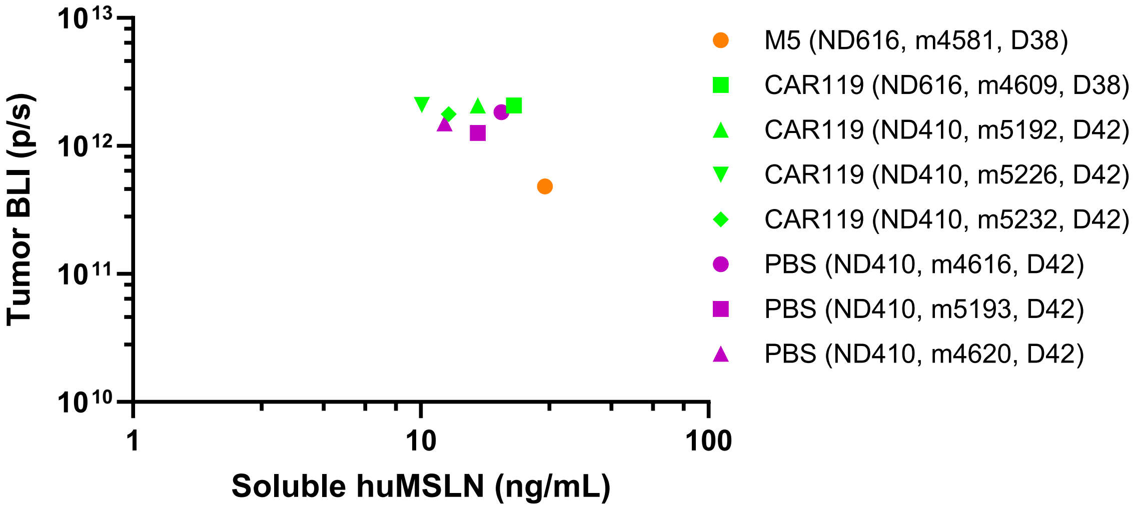

Supplementary Figure 11. Concentrations of soluble huMSLN detected in the ascites fluid plotted relative to tumor BLI. Ascites fluid was isolated from mice with uncontrolled tumors from ND616 and ND410 OvCAR8 studies presented in Figure 4d and soluble MSLN measured by ELISA. Figure legend indicates CAR treatment group (M5, 119, or PBS control) followed in parenthesis by the ND study, mouse number, and day of ascites isolation post T cell injection. Mice treated with CAR 422 and CAR 15B6 had low tumor BLI and no ascites. No soluble MSLN was detected in peritoneal washes of those mice.

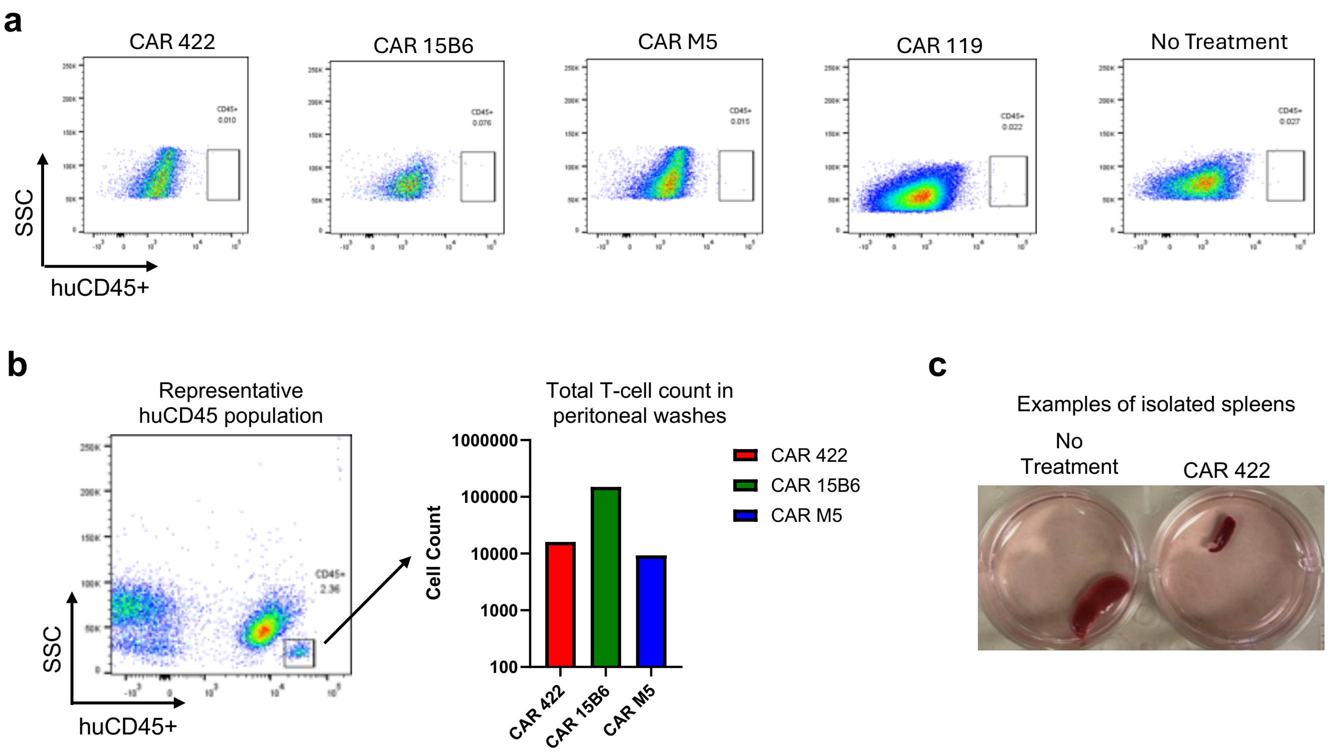

Supplementary Figure 12. Engraftment levels of CAR T cells in the OVCAR8 peritoneal mouse model. After tumors had cleared by Day 38 post treatment with CAR 422 and CAR 15B6 in the OVCAR8 experiment (Figure 4c, d), mice from ND616 study group (3 mice treated with each CAR-T) were sacrificed, and their blood and peritoneal washes were evaluated for the presence of human T cells by flow cytometry. **(a)** Examination of peripheral blood revealed absence of human T cells. **(b)** Examination of peritoneal washes revealed migration of human T cells into the peritoneum. **(c)** Representative images of mouse spleens showing a consistently smaller size in MSLN-targeting CARs than in CAR119 and no treatment control group.

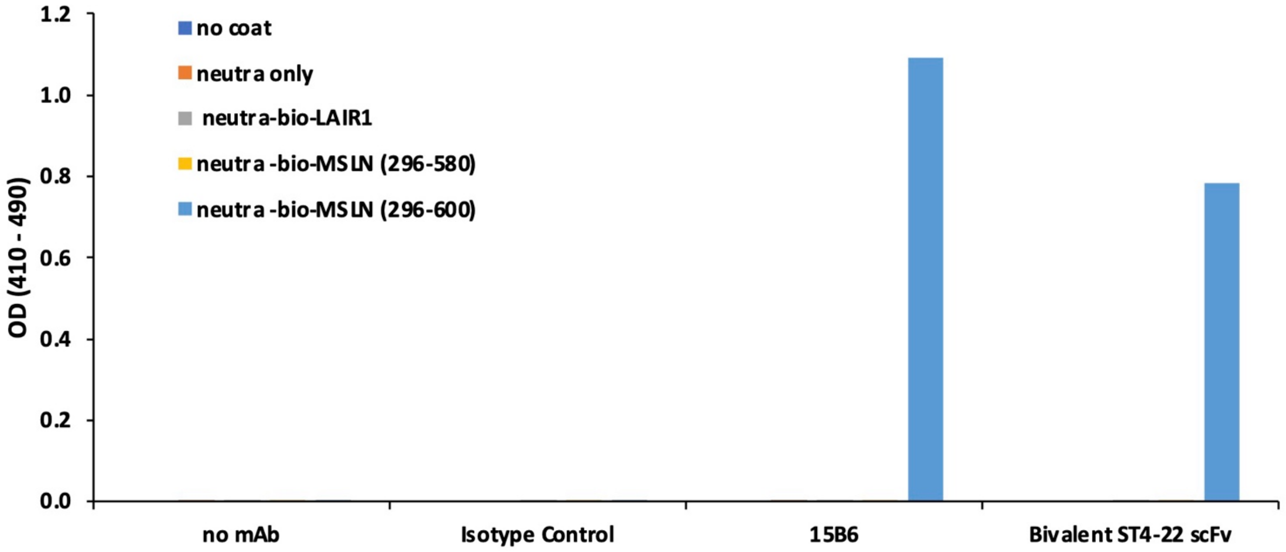

Supplementary Figure 13. Binding of bivalent ST422 scFv to MSLN polypeptides. A bivalent scFv form of ST4-22 produced to assess off-target binding in a membrane proteome array was first validated for its binding to MSLN (296-600) and MSLN (296-580) polypeptides representing full-length and shed domains, respectively. Controls included uncoated microplate wells and wells coated with only neutravidin (neutra) or neutravidin and biotinylated human LAIR1 protein. Bivalent ST4-22 svFv along with an isotype control (murine anti-STEAP2) and reference antibody 15B6 were applied to wells at 2000 ng/ml. Bivalent ST4-22 scFv binds only to full-length MSLN but not to polypeptides representing shed MSLN as expected.

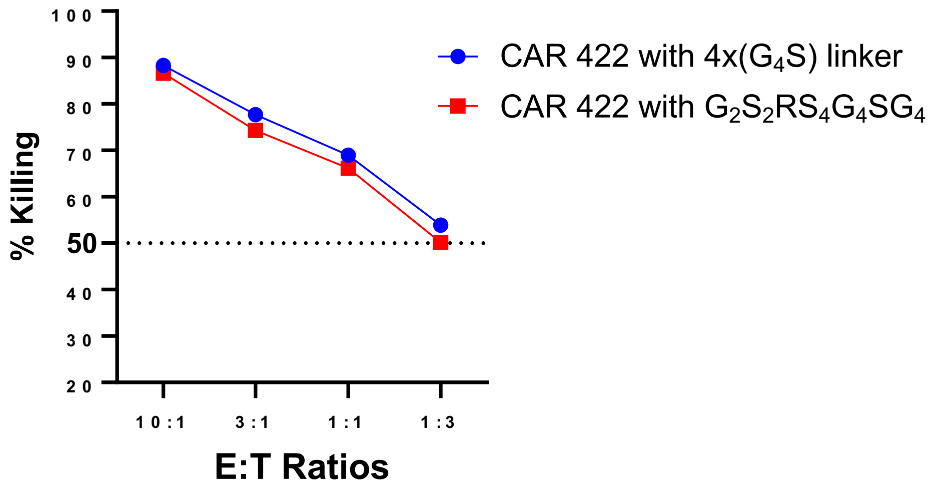

Supplementary Figure 14. Effect of light and heavy chain variable region linker length and composition on CAR 422 cytotoxicity. CAR 422 scFv 20-mer linker $G_2S_2RS_4G_4SG_4$ is compared 4x(G_4S) to assess impact on performance. In Incucyte® live cytotoxicity assays, CAR+ T cells were evaluated for % killing of AsPC1 cells as measured by loss of tumor GFP intensity at E:T ratios of 10, 3, 1 and .0.3 for 5 days. Percent killing on Day 5 is shown.
